# Symplectic Geometry of Teichmüller Spaces for Surfaces with Ideal Boundary

**DOI:** 10.1007/s00220-024-05075-7

**Published:** 2024-09-14

**Authors:** Anton Alekseev, Eckhard Meinrenken

**Affiliations:** 1 Section de Mathématiques, Université de Genéve, Genéve, Suisse; 2 Department of Mathematics, University of Toronto, 40 St. George Street, Toronto, Ontario M5S 2E4 Canada

## Abstract

A hyperbolic 0-metric on a surface with boundary is a hyperbolic metric on its interior, exhibiting the boundary behavior of the standard metric on the Poincaré disk. Consider the infinite-dimensional Teichmüller spaces of hyperbolic 0-metrics on oriented surfaces with boundary, up to diffeomorphisms fixing the boundary and homotopic to the identity. We show that these spaces have natural symplectic structures, depending only on the choice of an invariant metric on $\mathfrak {sl}(2,\mathbb {R})$. We prove that these Teichmüller spaces are Hamiltonian Virasoro spaces for the action of the universal cover of the group of diffeomorphisms of the boundary. We give an explicit formula for the Hill potential on the boundary defining the moment map. Furthermore, using Fenchel–Nielsen parameters we prove a Wolpert formula for the symplectic form, leading to global Darboux coordinates on the Teichmüller space.

## Introduction

A hyperbolic structure on a compact, oriented surface $\Sigma$ without boundary may be described by an atlas with oriented charts taking values in the Poincaré disk $\mathbb {D}$, with constant transition functions given by orientation preserving isometries of $\mathbb {D}$. The same definition may be used for surfaces $\Sigma$ *with boundary*, using as the model space the *closed* Poincaré disk $\overline{\mathbb {D}}$. Given a hyperbolic structure, the interior of the surface acquires a hyperbolic metric, exhibiting the same boundary behaviour as the standard metric on the Poincaré disk. Metrics of this type are known as *conformally compact hyperbolic metrics* or *hyperbolic 0-metrics*. The boundary components are regarded as a boundary at infinity, called *ideal boundary*. One pictures $\Sigma$ as a surface with funnel ends, also known as *trumpets*: 
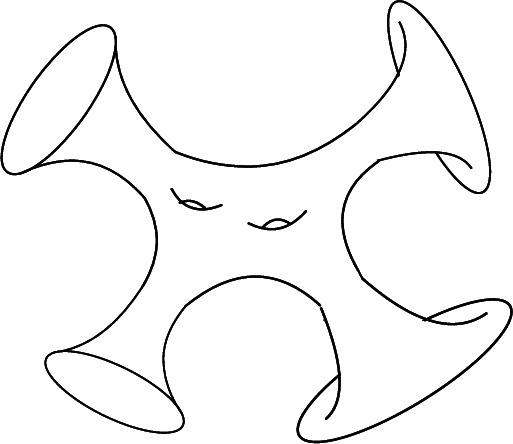


In this paper, we consider the Teichmüller space of hyperbolic structures,

$$\begin{aligned} {\text {Teich}}(\Sigma )={\text {Hyp}}(\Sigma )/ ^0{\text {Diff}}_\textsf{o}(\Sigma ), \end{aligned}$$

where $^0{\text {Diff}}_\textsf{o}(\Sigma )$ are the diffeomorphisms fixing the boundary and isotopic to the identity. If $\partial \Sigma \ne \emptyset$, the space ${\text {Teich}}(\Sigma )$ is infinite-dimensional. It has a residual action of the universal cover of the group ${\text {Diff}}_\textsf{o}(\partial {\Sigma })$ of orientation preserving diffeomorphisms of the boundary, and of the mapping class group ${\text {MCG}}(\Sigma )$. The infinite-dimensional Teichmüller space, and corresponding Riemann moduli space $\mathcal {M}(\Sigma )={\text {Teich}}(\Sigma )/{\text {MCG}}(\Sigma )$, have been studied in the literature from the perspective of complex geometry and quasi-conformal mappings. See for example Bers [5, Section 19], Thurston [34, Remark 4.6.17], and the work of Takhtajan-Teo [32, 33]; see Schippers-Staubach [28] for a comprehensive overview. However, it appears that the symplectic aspects of this space have been neglected. We will show:

### Theorem A

The space ${\text {Teich}}(\Sigma )$ has a natural (weak) symplectic structure, invariant under the action of the mapping class group and of the universal cover of ${\text {Diff}}_\textsf{o}(\partial {\Sigma })$.

The action of the universal cover of ${\text {Diff}}_\textsf{o}(\partial {\Sigma })$ admits a moment map, turning ${\text {Teich}}(\Sigma )$ into a Hamiltonian Virasoro space. Recall that the Virasoro Lie algebra $\mathfrak {vir}(\partial \Sigma )$ is a central extension of the Lie algebra ${\text {Vect}}(\partial {\Sigma })$ of vector fields on the boundary. Its smooth dual space comes with a map to $\mathbb {R}$, and the affine hyperplane $\mathfrak {vir}(\partial {\Sigma })^*_1$ at level 1 is identified with the space ${\text {Hill}}(\partial {\Sigma })$ of Hill operators on the boundary. Given a local coordinate *x* on the boundary, Hill operators are of the form $L=\frac{d^2}{d x^2}+T$ for a Hill potential *T*(*x*). The action of diffeomorphisms on Hill potentials *T*(*x*) is given by the expression

$$\begin{aligned} ({\textsf{F}}^{-1}. T)(x)={\textsf{F}}'(x)^2\,T({\textsf{F}}(x))+{\frac{1}{2}}\mathcal {S}({\textsf{F}})(x) \end{aligned}$$

involving the *Schwarzian derivative* $\mathcal {S}$.

### Theorem B

The action of the universal cover of ${\text {Diff}}_\textsf{o}(\partial {\Sigma })$ on ${\text {Teich}}(\partial {\Sigma })$ is Hamiltonian, with a canonically defined equivariant moment map $\Phi :{\text {Teich}}(\Sigma )\rightarrow {\text {Hill}}(\partial {\Sigma })^-$.

Here ${\text {Hill}}(\partial {\Sigma })^-=\mathfrak {vir}(\partial {\Sigma })^*_{-1}$ is the affine subspace at level $-1$ (consisting of all $-L$ where *L* is a Hill operator). The quickest description of the moment map is based on the observation that a hyperbolic structure on $\Sigma$ determines a projective structure on the boundary (by restricting the $\overline{\mathbb {D}}$-valued charts of $\Sigma$ to the boundary). As is well-known (see e.g. [25]), projective structures on oriented 1-manifolds are equivalent to Hill operators. The moment map takes $[\textsf{g}]$ to $-L$ where *L* is the Hill operator for the projective structure. For an explicit description, choose a local coordinate *x* on the boundary, and complete to a local coordinate system *x*, *y* where *y* is a boundary defining function. Let $a(x)>0$ be the positive function obtained from the most singular part of the Riemannian volume form: ${\textsf{d}} {\text {vol}}_{\textsf{g}}=y^{-2} a(x){\textsf{d}}x\wedge {\textsf{d}}y+O(y^{-1})$. Let *k*(*x*, *y*) be the geodesic curvature of the curve $(x,y)\mapsto (x+t,y)$; one finds $k(x,y)=1+c(x)y^2+O(y^3)$.

### Theorem C

The boundary Hill potential for the hyperbolic 0-metric $\textsf{g}$ is given by

$$\begin{aligned} T={\frac{1}{2}}\left( \frac{a''}{a}-\frac{3}{2}\Big (\frac{a'}{a}\Big )^2\right) +\frac{a^2}{2} c.\end{aligned}$$

If $\Sigma$ has negative Euler characteristic, we may use a pants decomposition to obtain a Fenchel–Nielsen parametrization

$$\begin{aligned} {\text {Teich}}(\Sigma )=(\mathbb {R}_{>0}\times \mathbb {R})^{3g-3+r}\times (\mathbb {R}_{>0}\times \widetilde{{\text {Diff}}}_\textsf{o}(S^1))^r. \end{aligned}$$

Let $(\ell _i,\tau _i)$ be the length and twist parameters for the first $3g-3+r$ factors (corresponding to gluing circles), and $\ell _j,{\textsf{F}}_j$ the parameters for the last *r* factors (corresponding to trumpet ends). We have the following version of Wolpert’s formula for the symplectic form (Theorems 8.3 and 8.2):

### Theorem D

In Fenchel–Nielsen parameters, the symplectic form on ${\text {Teich}}(\Sigma )$ is given by

$$\begin{aligned} \omega = {\frac{1}{2}}\sum _{i=1}^{3g-3+r} {\textsf{d}}\ell _i\wedge {\textsf{d}}\tau _i -\frac{1}{4}\sum _{j=1}^r{\textsf{d}}\int _{S^1}\big (\ell _j^2{\textsf{F}}'_j\,{\textsf{d}}{\textsf{F}}_j +({\textsf{F}}'_j)^{-1}{\textsf{d}}{\textsf{F}}''_j \big ) \end{aligned}$$

Here ${\textsf{d}}$ is the exterior differential on the diffeomorphism group, and $'$ denotes a derivative in the $S^1$-direction.

The terms in the second sum are symplectic forms on the factors $\mathbb {R}_{>0}\times \widetilde{{\text {Diff}}}_\textsf{o}(S^1)$, which may be interpreted as moduli spaces for the trumpet (with one geodesic end and one ideal boundary). We shall show (Proposition 8.5) how to introduce global Darboux coordinates for the trumpet moduli space, resulting in global Darboux coordinates for the space ${\text {Teich}}(\Sigma )$.

One of the motivations for this work are recent developments in the physics literature on Jackiw–Teitelboim gravity, notably the articles by Saad et al. [27], Maldacena et al. [20], Cotler et al. [7], and Stanford–Witten [31]. The discussion in these articles involves hyperbolic surfaces with funnel ends (‘trumpets’), using cut-off along ‘wiggly boundaries’ to create surfaces of finite volume, leading to a theory governed by a *Schwarzian action*. As shown by the physicists, this relates JT gravity to mathematical concepts such as the Mirzakhani recursion formulas for Weil–Petersson volumes, topological recursion and random matrix theory, Duistermaat–Heckman theory for Virasoro coadjoint orbits, and more.

A second motivation is our program to develop a theory of Hamiltonian Virasoro spaces, analogous to the theory of Hamiltonian loop group spaces [23]. An important example of such a space is the infinite dimensional moduli space

$$\begin{aligned} \mathcal {M}_G(\Sigma )=\frac{ \{ A \in \Omega ^1(\Sigma , \mathfrak {g})|\ {\textsf{d}}A+{\frac{1}{2}}[A,A]=0\}}{ \{g:\Sigma \rightarrow G :g|_{\partial \Sigma } =e\} } \end{aligned}$$

of flat *G*-connections, where *G* is a simply connected Lie group with an invariant metric on its Lie algebra. This space has a symplectic form given by reduction, and the residual action of ${\text {Map}}(\partial \Sigma ,G)$ is Hamiltonian, with affine moment map given by the pullback of the connection, $[A]\mapsto \iota _{\partial {\Sigma }}^*A$. It is natural to have a similar example for the Virasoro setting; in fact the two situations are related by Drinfeld–Sokolov reduction. As shown in [2], Hamiltonian loop group spaces with proper moment map are equivalent to finite-dimensional quasi-Hamiltonian *G*-spaces; the results of [3] pave the way for a similar correspondence for Hamiltonian Virasoro spaces.

Let us briefly summarize our construction of the symplectic form on ${\text {Teich}}(\Sigma )$. By a famous result of Goldman [13] and Hitchin [16], the Teichmüller space for a surface $\Sigma$ *without boundary*, of genus $\textsf{g}\ge 2$, is a moduli space $\mathcal {A}_{{\text {flat}}}(P)/ {\text {Gau}} (P)$ of flat connections on a principal *G*-bundle $P\rightarrow \Sigma$ of Euler number $2\textsf{g}-2$. In particular, the Weil-Petersson symplectic form is obtained by reduction of the Atiyah–Bott symplectic structure on the space of connections. There does not seem to be an immediate generalization of this result to the case of non-empty boundary. Instead, motivated by the theory of geometric structures [14] we take as our starting point is a principal *G*-bundle $P\rightarrow \Sigma$ together with a *G*-equivariant morphism $\sigma :P\rightarrow \overline{\mathbb {D}}$, called a *developing section*. For suitable choice of $(P,\sigma )$, we define

$$\begin{aligned} \widehat{{\text {Teich}}}(\Sigma )=\mathcal {A}_{{\text {flat}}}^{{\text {pos}}}(P)/ {\text {Aut}} _\textsf{o}(P,\partial P,\sigma ),\end{aligned}$$

the quotient of the space of flat connections satisfying a certain positivity condition with respect to $\sigma$, by the identity component of automorphisms preserving $\sigma$ and trivial along the boundary. (This space may be interpreted as elements of ${\text {Teich}}(\Sigma )$ together with developing sections for the projective structure on the boundary.) We show that this space is a symplectic quotient for the Atiyah–Bott symplectic structure on $\mathcal {A}^{{\text {pos}}}(P)$. It comes with a residual action of ${\text {Gau}} (\partial P,\partial \sigma )$, the gauge transformations of $\partial P=P|_{\partial {\Sigma }}$ preserving $\partial \sigma =\sigma |_{\partial P}$. We prove that the latter action is Hamiltonian, with moment map image a single coadjoint orbit $\mathcal {O}$, and(a symplectic reduction). This defines the symplectic structure on ${\text {Teich}}(\Sigma )$ (Theorem A). The moment map (Theorem B) is obtained by explicit calculation, beginning with the moment map for the action of the full group of automorphisms on the space $\mathcal {A}(P)$. The relevant background material on the Atiyah–Bott construction is provided in the “Appendix”.

The structure of the paper is as follows. In Sect. 2, we recall basic definitions and properties of hyperbolic structures on surfaces with boundary, and introduce infinite dimensional Teichmüller spaces $\textrm{Teich}(\Sigma )$. In Sect. 3, using the moving coframe formalism of É. Cartan, we describe the relation between hyperbolic metrics and flat $\mathfrak {sl}(2, \mathbb {R})$ connection 1-forms. We prove local normal forms for coframes; as a by-product this gives a new proof of the local normal form for hyperbolic 0-metrics near the ideal boundary. In Sect. 4, we use a more global approach, considering principal $\textrm{PSL}(2, \mathbb {R})$ bundles $P\rightarrow \Sigma$ equipped with a developing section $\sigma$. For suitable choice of *P*, we exhibit ${\text {Teich}}(\Sigma )$ as a space of $\sigma$-positive flat connections modulo a subgroup of bundle automorphisms preserving $\sigma$. Section 5 is devoted to a detailed study of this group of automorphisms. In Sect. 6, we apply the Atiyah–Bott construction to the space of positive connections, and explain how to obtain $\textrm{Teich}(\Sigma )$ by reduction. In Sect. 7, we prove that the diffeomorphisms of the boundary act on $\textrm{Teich}(\Sigma )$, that this action is Hamiltonian, and that it corresponds to the Virasoro central extension of the diffeomorphism group. In Sect. 8, we give an explicit description of the symplectic structure in Fenchel–Nielsen parameters, and give a construction of Darboux coordinates on $\textrm{Teich}(\Sigma )$ which combines the classical Wolpert formula [37] with the construction of Darboux coordinates on hyperbolic Virasoro coadjoint orbits [1].

## Hyperbolic Structures on Surfaces with Boundary

### Hyperbolic and projective structures

The model space for hyperbolic structures on surfaces $\Sigma$ without boundary is the Poincaré disk

$$\begin{aligned} \mathbb {D}=\{z\in \mathbb {C}|\ |z|<1\}, \end{aligned}$$

with the action of $G={\text {PSU}}(1,1)$ by Möbius transformations. The *G*-action on $\mathbb {D}$ extends to the closed Poincaré disk $\overline{\mathbb {D}}$; this will be our model space for surfaces with boundary:

#### Definition 2.1

A *hyperbolic structure* on an oriented surface $\Sigma$ with boundary $\partial \Sigma$ is an oriented atlas with $\overline{\mathbb {D}}$-valued charts, with constant transition maps given by elements of *G*. The space of all hyperbolic structures on $\Sigma$ will be denoted ${\text {Hyp}}(\Sigma )$.

#### Remark 2.2

A hyperbolic structure on $\Sigma$ pulls back to a hyperbolic structure on every covering space of $\Sigma$. If $\Sigma$ is compact and connected, then the universal covering space is of the form $\overline{\mathbb {D}}-\mathfrak {L}$ where $\mathfrak {L}\subseteq \partial \mathbb {D}$ is a set of *limit points*, and

1 $$\begin{aligned} \Sigma =(\overline{\mathbb {D}}-\mathfrak {L})/\Gamma \end{aligned}$$

where $\Gamma \subseteq G$ is a Fuchsian group of Schottky type. See [6] or [30].

In a similar way, taking the boundary of the Poincaré disk as the model space for projective structures, we define:

#### Definition 2.3

A *projective structure* on an oriented 1-manifold $\textsf{C}$ (without boundary) is an oriented atlas with $\partial \mathbb {D}$-valued charts, with constant transition maps given by elements of *G*. The space of all projective structures on $\textsf{C}$ will be denoted ${\text {Proj}}(\textsf{C})$.

A hyperbolic structure on $\Sigma$ determines a projective structure on the boundary – one simply restricts the $\overline{\mathbb {D}}$-valued charts. This gives a canonical map

2 $$\begin{aligned} {\text {Hyp}}(\Sigma )\rightarrow {\text {Proj}}(\partial {\Sigma }). \end{aligned}$$

### Hyperbolic 0-metrics

The $G={\text {PSU}}(1,1)$-action on the Poincaré disk preserves the Poincaré metric, written in polar coordinates $z=r e^{i\varphi }$ as

3 $$\begin{aligned} \frac{4}{(1-r^2)^2}({\textsf{d}}r^2+r^2{\textsf{d}}\varphi ^2). \end{aligned}$$

Hence, a hyperbolic structure on $\Sigma$ determines a Riemannian metric $\textsf{g}$ on the interior ${\text {int}}(\Sigma )$, by pulling back (3) under the coordinate charts. At the boundary $\partial \Sigma$, this metric becomes singular in the same way as the metric on $\overline{\mathbb {D}}$: letting $\varrho$ be any boundary defining function, the product $\varrho ^2 \textsf{g}$ extends to an ordinary metric on $\Sigma$. Riemannian metrics with this property may be seen as ordinary Euclidean metrics on the *0-tangent bundle* of Mazzeo–Melrose [21, 22], i.e., the Lie algebroid

$$\begin{aligned}  ^0T\Sigma \rightarrow \Sigma \end{aligned}$$

whose sections are the vector fields on $\Sigma$ that vanish along the boundary. A given *0-metric* is called hyperbolic if its restriction to the interior is hyperbolic in the sense that it has Gauss curvature $K_{\textsf{g}}=-1$. Theorem 3.9 below says that all hyperbolic 0-metrics arise from hyperbolic structures as in Definition 2.1.

### Teichmüller spaces

For groups of diffeomorphisms of a compact, oriented manifold, we use a subscript $+$ to indicate diffeomorphisms preserving orientation, and subscript $\textsf{o}$ to indicate diffeomorphisms isotopic to the identity. We recall that the universal cover of ${\text {Diff}}_+(S^1)={\text {Diff}}_\textsf{o}(S^1)$ is identified with $\mathbb {Z}$-equivariant diffeomorphisms of $\mathbb {R}$.

Let $\Sigma$ be compact, connected, and oriented. Denote by $^b {\text {Diff}}(\Sigma )$ the diffeomorphisms preserving the boundary, and by $^0{\text {Diff}}(\Sigma )$ the subgroup of diffeomorphisms fixing the boundary pointwise. We hence have subgroups

4 $$\begin{aligned}  ^b {\text {Diff}}(\Sigma )\supseteq \! ^b {\text {Diff}}_+(\Sigma )\supseteq \! ^b {\text {Diff}}_\textsf{o}(\Sigma ) \end{aligned}$$

and similarly for $^0{\text {Diff}}(\Sigma )$. (The superscripts 0, *b* are omitted if $\partial {\Sigma }=\emptyset$.) The mapping class group is the quotient ${\text {MCG}}(\Sigma )= ^0{\text {Diff}}_+(\Sigma )/ ^0{\text {Diff}}_\textsf{o}(\Sigma )$.

#### Definition 2.4

The (infinite-dimensional) *Teichmüller space* is the space of hyperbolic structures on $\Sigma$, up to diffeomorphisms fixing the boundary and homotopic to the identity:

5 $$\begin{aligned} {\text {Teich}}(\Sigma )={\text {Hyp}}(\Sigma )/ ^0{\text {Diff}}_\textsf{o}(\Sigma ). \end{aligned}$$

The (infinite-dimensional) (Riemann) *moduli space* is the quotient

6 $$\begin{aligned} \mathcal {M}(\Sigma )={\text {Hyp}}(\Sigma )/ ^0{\text {Diff}}_+(\Sigma ). \end{aligned}$$

Equivalently, $\mathcal {M}(\Sigma )$ is the quotient of ${\text {Teich}}(\Sigma )$ under the action of the mapping class group. Both (5) and (6) are endowed with residual actions of boundary diffeomorphisms. Since $^b {\text {Diff}}_\textsf{o}(\Sigma )/ ^0{\text {Diff}}_\textsf{o}(\Sigma )={\text {Diff}}_\textsf{o}(\partial {\Sigma })$, there is an induced action

$$\begin{aligned} {\text {Diff}}_\textsf{o}(\partial {\Sigma })\circlearrowright \mathcal {M}(\Sigma ). \end{aligned}$$

Similarly, the quotient $^b {\text {Diff}}_\textsf{o}(\Sigma )/ ^0{\text {Diff}}_\textsf{o}(\Sigma )$ acts on ${\text {Teich}}(\Sigma )$. This group is a covering of ${\text {Diff}}_\textsf{o}(\partial {\Sigma })$ (not always the universal cover; see examples below). In any case, there is an induced action

$$\begin{aligned} \widetilde{{\text {Diff}}}_\textsf{o}(\partial {\Sigma })\circlearrowright {\text {Teich}}(\Sigma ) \end{aligned}$$

of the universal cover of the identity component ${\text {Diff}}_\textsf{o}(\partial {\Sigma })$. (If $\partial {\Sigma }$ has several components, we may also consider diffeomorphisms interchanging boundary components.) The map (2) descends to maps

7 $$\begin{aligned} {\text {Teich}}(\Sigma )\rightarrow {\text {Proj}}(\partial {\Sigma }),\ \ \mathcal {M}(\Sigma ) \rightarrow {\text {Proj}}(\partial {\Sigma }), \end{aligned}$$

which are equivariant for these actions. As we shall explain in this paper, these will be identified as moment maps for Hamiltonian Virasoro spaces.

### Special cases

One has more concrete descriptions of the Teichmüller spaces, as follows. Let *g* be the genus of $\Sigma$ and *r* the number of boundary components. The Euler characteristic is thus $\chi (\Sigma )=2-2g-r$.

#### Poincaré disk

($$g=0,\ r=1$$.)

The standard metric on the closed Poincaré disk $\overline{\mathbb {D}}$ is the unique hyperbolic 0-metric on the disk, up to diffeomorphism. The stabilizer of the standard metric under the action of $^b {\text {Diff}}_+(\overline{\mathbb {D}})$ is $G={\text {PSU}}(1,1)$. It follows that ${\text {Hyp}}(\overline{\mathbb {D}})= ^b {\text {Diff}}_+(\overline{\mathbb {D}})/G$, and hence

$$\begin{aligned} {\text {Teich}}(\overline{\mathbb {D}})=\mathcal {M}(\overline{\mathbb {D}})={\text {Diff}}_\textsf{o}(\partial \mathbb {D})/G \end{aligned}$$

(since every diffeomorphism in $^0{\text {Diff}}_+(\overline{\mathbb {D}})$ is isotopic to the identity). This space is closely related to Bers’ *universal Teichmüller space* [4, 32]; it has an interpretation as a coadjoint orbit of the Virasoro group.

#### Hyperbolic cylinders

($$g=0,\ r=2$$.)

Let $\mathbb {A}=S^1\times (-\infty ,\infty )$ be the infinite cylinder (open annulus), with coordinates (*x*, *u*) where $x\in S^1=\mathbb {R}/\mathbb {Z}$. Denote by $\overline{\mathbb {A}}=S^1\times [-\infty ,\infty ]$ its compactification, with boundary defining function $\varrho (x,u)=\cosh (u)^{-1}$. Here ${\text {MCG}}(\overline{\mathbb {A}})=\mathbb {Z}$, generated by Dehn twists $(x,u)\mapsto (x+f(u),u)$ for $f\in C^\infty (\mathbb {R})$ with $f(u)=0$ for $u<-R$ and $f(u)=1$ for $u>R$, for some $R>0$.

Given $\ell >0$, the formula

8 $$\begin{aligned} \textsf{g}= \cosh ^2(u) \ell ^2 {\textsf{d}}x^2 +{\textsf{d}}u^2 \end{aligned}$$

defines a hyperbolic 0-metric, with the curve $u=0$ as its unique closed simple geodesic. 
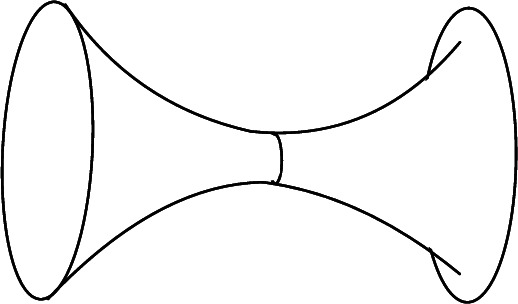
 Conversely, every hyperbolic 0-metric on $\overline{\mathbb {A}}$ admits a unique closed simple geodesic; letting $\ell$ be its length, the metric is obtained from (8) by the action of an element of ${\text {Diff}}_+(\overline{\mathbb {A}})$ preserving the two boundary components. The stabilizer of (8) under this action is $S^1$, acting by rotations of the cylinder. This gives

$$\begin{aligned} \mathcal {M}(\overline{\mathbb {A}})= \mathbb {R}_{>0}\times ({\text {Diff}}_\textsf{o}(S^1)\times {\text {Diff}}_\textsf{o}(S^1))/S^1 \end{aligned}$$

with the anti-diagonal embedding of $S^1$. For the Teichmüller space, we note that compactly supported diffeomorphisms of $(-\infty ,0]\times S^1$, modulo the subgroup fixing the boundary, is the universal cover $\widetilde{{\text {Diff}}}_\textsf{o}(S^1)$. This gives

$$\begin{aligned} {\text {Teich}}(\overline{\mathbb {A}})=\mathbb {R}_{>0}\times (\widetilde{{\text {Diff}}}_\textsf{o}(S^1)\times \widetilde{{\text {Diff}}}_\textsf{o}(S^1) )/\mathbb {R}\end{aligned}$$

where $\mathbb {R}$ is embedded anti-diagonally. For $\overline{\mathbb {A}}$ with the 0-metric (8), the subset given by $u\ge 0$ is called a *trumpet* (also called *flare* or *funnel*). It has one geodesic boundary component and one boundary at infinity.

#### Surfaces of negative Euler characteristic ($g=0,\ r>2$ or $g\ge 1,\ r\ge 1$)

After choice of a Fenchel–Nielsen parametrization, one finds

9 $$\begin{aligned} {\text {Teich}}(\Sigma )\cong (\mathbb {R}_{>0}\times \mathbb {R})^{3g-3+r}\times \prod _{j=1}^r (\mathbb {R}_{>0}\times \widetilde{{\text {Diff}}}_\textsf{o}(S^1)). \end{aligned}$$

We will give details in Sect. 8 below. At this point, we just mention that the Fenchel–Nielsen parametrization involves a pants decomposition of the surface. The boundaries of the pants which are not among the boundaries of $\Sigma$ form a system of $3g-3+r$ circles separating the pants; given a hyperbolic 0-metric these are realized as geodesics, and the factors $\mathbb {R}_{>0}\times \mathbb {R}$ in (9) are the corresponding length and twist parameters. The boundary components of pants which are also boundaries of the surface correspond to trumpets as discussed in Sect. 2.4.2; the $\mathbb {R}_{>0}\times \widetilde{{\text {Diff}}}_\textsf{o}(S^1)$-factors signify the length of the geodesic end of the trumpet and a twist by rotating the ideal boundary of the trumpet.

## Coframe Formalism

For calculations in this section, we will prefer the half-plane model of hyperbolic geometry. Let

$$\begin{aligned} \mathbb {H}=\{z=x+iy| y>0\},\end{aligned}$$

with the standard hyperbolic metric

10 $$\begin{aligned} \frac{1}{y^2}({\textsf{d}}x^2+{\textsf{d}}y^2). \end{aligned}$$

The map $\mathbb {H}\rightarrow \mathbb {D},\ z\mapsto (z-i)/(1-iz)$ is an isometric isomorphism, equivariant with respect to

11 $$\begin{aligned} {\text {Ad}} _q:{\text {PSL}}(2,\mathbb {R})\rightarrow {\text {PSU}}(1,1),\ \ \ q= \Big [\begin{array}{cc} 1&  -i\\ -i&  1\end{array}\Big ]. \end{aligned}$$

Here the bracket notation $[A]\in {\text {PGL}}(2,\mathbb {C})$ denotes the image of an element $A\in {\text {GL}}(2,\mathbb {C})$. The isomorphism extends to a bijection $\overline{\mathbb {H}}\cup \{\infty \}\rightarrow \overline{\mathbb {D}}$ taking $0\in \partial \mathbb {H}$ to $-i\in \partial \mathbb {D}$. Throughout this section, *G* denotes the group ${\text {PSL}}(2,\mathbb {R})$, and $\mathfrak {g}$ its Lie algebra $\mathfrak {sl}(2,\mathbb {R})$.

### Cartan coframe formalism

A convenient tool for dealing with 0-metrics is the *moving coframe formalism* due to É. Cartan. Let $\Sigma$ be an oriented surface with boundary. Let $^0\Omega ^k(\Sigma )=\Gamma (\bigwedge ^k\,( ^0T\Sigma )^*)$ denote the de Rham complex of the Lie algebroid $^0T\Sigma$. Elements of this space may be seen as ordinary *k*-forms on ${\text {int}}(\Sigma )$ such that $\varrho ^k\alpha$ extends to all of $\Sigma$, for any boundary defining function $\varrho$.

An *oriented coframe* over an open subset $U\subseteq \Sigma$ is pair of 0-covector fields

$$\begin{aligned}\alpha _1,\alpha _2\in  ^0\Omega ^1(U)\end{aligned}$$

giving an oriented frame for $( ^0T \Sigma )^*|_U$. Given a 0-metric $\textsf{g}$ on $\Sigma$, the coframe is *orthonormal* if

$$\begin{aligned} \textsf{g}|_U=(\alpha _1)^2+(\alpha _2)^2. \end{aligned}$$

In this case, the Riemannian volume form ${\textsf{d}} {\text {vol}}_{\textsf{g}} \in  ^0\Omega ^2(\Sigma )$ is given by

$$\begin{aligned} {\textsf{d}} {\text {vol}}_{\textsf{g}}|_U =\alpha _1\wedge \alpha _2. \end{aligned}$$

Any two oriented orthonormal coframes for $\textsf{g}$ are related, on the overlap of their domains, by a *coframe rotation*

12 $$\begin{aligned} \alpha _1'=\cos \varphi \alpha _1+\sin \varphi \alpha _2,\ \ \alpha _2'=-\sin \varphi \alpha _1+\cos \varphi \alpha _2 \end{aligned}$$

with $\varphi \in C^\infty (U\cap U',\mathbb {R})$. The *spin connection* for an oriented coframe is the 0-covector field

$$\begin{aligned} \kappa \in  ^0\Omega ^1(U) \end{aligned}$$

defined by the equations ${\textsf{d}}\alpha _1=-\kappa \wedge \alpha _2,\ \ {\textsf{d}}\alpha _2=\kappa \wedge \alpha _1.$ Under coframe rotation, the spin connection changes to $\kappa '=\kappa -{\textsf{d}}\varphi$. This shows that there exists a globally defined function $K_{\textsf{g}}\in C^\infty (\Sigma )$ such that

$$\begin{aligned} K_{\textsf{g}}\, {\textsf{d}} {\text {vol}}_{\textsf{g}}\big |_U={\textsf{d}}\kappa .\end{aligned}$$

This function is the *Gauss curvature* of the metric. The three equations

13 $$\begin{aligned} {\textsf{d}}\alpha _1=-\kappa \wedge \alpha _2,\ \ {\textsf{d}}\alpha _2=\kappa \wedge \alpha _1,\ \ {\textsf{d}}\kappa =K_{\textsf{g}}\ \alpha _1\wedge \alpha _2 \end{aligned}$$

are *Cartan’s structure equations*.

#### Examples 3.1

We list some standard coframes for hyperbolic 0-metrics, and the resulting spin connections.

- Upper half plane $\bar{\mathbb {H}}$:
  $$\begin{aligned} \alpha _1=\frac{{\textsf{d}}x}{y},\ \alpha _2=\frac{{\textsf{d}}y}{y},\ \kappa =-\frac{{\textsf{d}}x}{y}.\end{aligned}$$
- Poincare disk $$\overline{\mathbb {D}}$$:
  $$\begin{aligned} \alpha _1=\frac{2}{1-r^2} r{\textsf{d}}\varphi ,\ \ \alpha _2=\frac{2}{1-r^2} {\textsf{d}}r,\ \ \kappa =\frac{1+r^2}{1-r^2}{\textsf{d}}\varphi .\end{aligned}$$
  Replacing *r* with the boundary defining function $y=\frac{1-r}{1+r}$, this becomes
  $$\begin{aligned} \alpha _1=\frac{1-y^2}{2} \frac{{\textsf{d}}\varphi }{y},\ \ \alpha _2=\frac{{\textsf{d}}y}{y},\ \ \kappa =-\frac{1+y^2}{2} \frac{{\textsf{d}}\varphi }{y}\end{aligned}$$
  which more clearly exhibits the coframe as 0-covector fields.
- Hyperbolic cylinder $\overline{\mathbb {A}}=S^1\times [\infty ,\infty ]$ with parameter $\ell >0$: Using coordinates *x*, *u*,
  $$\begin{aligned} \alpha _1=\cosh (u)\ell \ {\textsf{d}}x,\ \ \alpha _2=-{\textsf{d}}u,\ \ \kappa =-\sinh (u)\ell {\textsf{d}}x.\end{aligned}$$
  Putting $y=e^{-u}$ (which is a boundary defining function near the boundary $u=\infty$), this becomes
  $$\begin{aligned} \alpha _1=\ell \frac{1+y^2}{2}\ \frac{{\textsf{d}}x}{y},\ \ \alpha _2=\frac{{\textsf{d}}y}{y},\ \ \kappa =-\ell \frac{1-y^2}{2}\ \frac{{\textsf{d}}x}{y}. \end{aligned}$$
- Fefferman–Graham coframe: For any function *T*(*x*), the formulas
  $$\begin{aligned} \alpha _1=\big (1-y^2 T(x)\big ) \frac{{\textsf{d}}x}{y},\ \ \alpha _2=\frac{{\textsf{d}}y}{y},\ \ \kappa =-\big (1+y^2 T(x)\big ) \frac{{\textsf{d}}x}{y} \end{aligned}$$
  define a coframe for a hyperbolic 0-metric on $\{(x,y)|\ y\ge 0,\ y^2 T(x)<1\}$. This unifies the boundary behaviour of the previous examples.

#### Remark 3.2

From now on, the letters *x*, *y* will be reserved for local coordinates on $U\subseteq \Sigma$ that are *oriented* (i.e., ${\textsf{d}}x\wedge {\textsf{d}}y>0$) and *adapted to the boundary*, in the sense that *y* is a boundary defining function for $\partial \Sigma \cap U$. If *U* is contained in the interior, this just means $y>0$ everywhere on *U*.

### The connection 1-form associated with an orthonormal coframe

Given a local orthonormal coframe $\alpha _1,\alpha _2\in  ^0\Omega ^1(U)$ for a 0-metric $\textsf{g}$, with associated spin connection $\kappa$, define a 0-connection 1-form

14 $$\begin{aligned} A={\frac{1}{2}}\left( \begin{array}{cc} \alpha _2 &  \alpha _1-\kappa \\ \alpha _1+\kappa &  -\alpha _2\end{array}\right) \in  ^0\Omega ^1(U,\mathfrak {g}).\end{aligned}$$

The curvature $F_A={\textsf{d}}A+{\frac{1}{2}}[A,A]$ is given by

15 $$\begin{aligned} F_A= (K_\textsf{g}+1) \left( \begin{array}{cc} 0 &  -1\\ 1 &  0 \end{array} \right) \ {\textsf{d}} {\text {vol}}_{\textsf{g}}.\end{aligned}$$

In particular, the 0-metric $\textsf{g}$ is hyperbolic if and only if the connection 1-form *A* is flat. Let $K\subseteq G={\text {PSL}}(2,\mathbb {R})$ be the maximal compact subgroup given as the stabilizer of $i\in \mathbb {H}$. It is identified with ${\text {SO}}(2)$ by the map

16 $$\begin{aligned} {\text {SO}}(2)\rightarrow K,\ \ R(\varphi )=\left( \begin{array}{cc} \cos (\varphi ) &  -\sin (\varphi )\\ \sin (\varphi )&  \cos (\varphi )\end{array}\right) \mapsto [R(\varphi /2)]. \end{aligned}$$

Under coframe rotations (12), the 0-connection 1-form *A* transforms by

17 $$\begin{aligned} A'=[R\left( \varphi /2\right) ]\bullet A\end{aligned}$$

where $\bullet$ signifies a gauge transformation

$$\begin{aligned} g\bullet A= {\text {Ad}} _g(A)-({\textsf{d}}g) g^{-1}\end{aligned}$$

for $g\in C^\infty (U,G)$. We hence see that the *K*-action on connection 1-forms translates into the ${\text {SO}}(2)$-action on coframes. Observe that *A* does *not* transform as a connection on the tangent bundle.

#### Example 3.3

For the upper half plane $\bar{\mathbb {H}}$ with its standard coframe (Example 3.1),

$$\begin{aligned} A= \left( \begin{array}{cc} \frac{1}{2y}\ {\textsf{d}}y &  \frac{1}{y}{\textsf{d}}x\\ 0 &  -\frac{1}{2y}\ {\textsf{d}}y\end{array}\right) .\end{aligned}$$

Note that $A= g^{-1}\bullet 0$ where

$$\begin{aligned} g=\left( \begin{array}{cc} 1 &  x \\ 0 &  1 \end{array}\right) \left( \begin{array}{cc} y^{\frac{1}{2}} &  0 \\ 0 &  y^{-\frac{1}{2}} \end{array}\right) . \end{aligned}$$

#### Example 3.4

(Fefferman–Graham gauge) The 0-connection form defined by the Fefferman–Graham coframe (Example 3.1d) reads as

$$\begin{aligned} A= \left( \begin{array}{cc} \frac{1}{2y}\ {\textsf{d}}y &  \frac{1}{y} {\textsf{d}}x\\ -T(x)y{\textsf{d}}x&  -\frac{1}{2y}\ {\textsf{d}}y\end{array}\right) . \end{aligned}$$

### Adapted coframes

For any manifold *M* with boundary $\partial M$, the restriction of the 0-cotangent bundle to the boundary has a distinguished trivial subbundle

$$\begin{aligned} \partial M\times \mathbb {R}\subseteq ( ^0TM)^*|_{\partial M},\end{aligned}$$

spanned by the restriction of $\varrho ^{-1}{\textsf{d}}\varrho \in  ^0\Omega ^1(M)$, for any boundary defining function $\varrho$. (Changing $\varrho$ by a positive function changes this expression by an *ordinary* (exact) 1-form, but the restriction of a regular 1-form as a section of the 0-tangent bundle vanishes.)

#### Definition 3.5

An oriented coframe $\alpha _1,\alpha _2\in  ^0\Omega ^1(U)$, defined on a neighborhood $U\subseteq \Sigma$ of the boundary, is *adapted to the boundary* if $\alpha _2|_{\partial {\Sigma }}$ is the canonical section of $( ^0T\Sigma )^*|_{\partial {\Sigma }}$. That is,

$$\begin{aligned} \alpha _2=\frac{d \varrho }{\varrho }+O(\varrho ^0).\end{aligned}$$

Here we write $\alpha =O(\varrho ^k)$ if $\varrho ^{-k}\alpha$ extends smoothly to the boundary (as an ordinary differential form). Note that for an adapted coframe, $\varrho \alpha _1$ pulls back to a volume form on $\partial \Sigma$.

The coframes in parts (a), (b), (d) of Examples 3.1 are adapted to the boundary. (But (b) is not defined at the center of $\overline{\mathbb {D}}$.) The coframe in (c) for the double trumpet is adapted to the boundary at $u=\infty$ but not at $u=-\infty$. One can turn it into an adapted coframe by applying a coframe rotation $R(\phi$ with $\phi |_{u=-\infty }=\pi ,\ \phi |_{u=0}=0$. If $\Sigma$ is compact and connected, of non-zero Euler characteristic, it is impossible, by Poincaré’s theorem on zeroes of vector fields on surfaces, to find a *global* oriented coframe that is adapted to all the boundary components. On the other hand, we have:

#### Lemma 3.6

Every hyperbolic 0-metric $\textsf{g}$ admits an oriented orthonormal coframe $\alpha _1,\alpha _2$ on some collar neighborhood of the boundary, which is adapted to the boundary. The spin connection of such a coframe satisfies

$$\begin{aligned} \kappa =-\alpha _1+O(\varrho ^1). \end{aligned}$$

#### Proof

Choose an oriented orthonormal coframe $\alpha _1,\alpha _2\in  ^0\Omega (U)$ on a collar neighborhood *U* of the boundary, with the property that $\alpha _2|_{\partial \Sigma }$ is a positive function times the canonical section of $( ^0T\Sigma )^*|_{\partial {\Sigma }}$. Using that $\textsf{g}$ is hyperbolic, we shall show that $\alpha _2|_{\partial \Sigma }$ must then be *equal to* the canonical section. It suffices to prove this in local coordinates *x*, *y* adapted to the boundary (cf.  Remark 3.2). Consider the Laurent expansions in powers of *y*,

$$\begin{aligned} \alpha _1=\frac{1}{y}\beta _1+\gamma _1+O(y^1),\ \ \alpha _2=\frac{h}{y}{\textsf{d}}y+\gamma _2+O(y^1),\ \ \kappa =\frac{1}{y}\beta _3+\gamma _3+O(y^1), \end{aligned}$$

where $\beta _i,\gamma _i$, are of the form $f_i(x){\textsf{d}}x+g_i(x)\ {\textsf{d}}y$, and where *h* is a function of *x*, with $h(x)>0$. Comparing coefficients of $y^{-2}$ in the structure equations (13) (with $K_{\textsf{g}}=-1$) gives conditions

18 $$\begin{aligned} \beta _1\wedge {\textsf{d}}y=-h\beta _3\wedge {\textsf{d}}y,\ \ 0=\beta _3\wedge \beta _1,\ \ \beta _3\wedge {\textsf{d}}y=-h\beta _1\wedge {\textsf{d}}y. \end{aligned}$$

From

$$\begin{aligned} {\textsf{d}} {\text {vol}}_\textsf{g}=\alpha _1\wedge \alpha _2=\frac{1}{y^2}h \beta _1\wedge {\textsf{d}}y+O(y^{-1}) \end{aligned}$$

we see that $\beta _1,{\textsf{d}}y$ are pointwise linearly independent; in particular $\beta _1$ is non-vanishing. Hence, the second equation in (18) shows that $\beta _3$ is a scalar multiple of $\beta _1$, and so the other two equations give $\beta _1=-h\beta _3,\ \beta _3=-h\beta _1$. Hence $h=1$ and $\beta _3=-\beta _1$. At this stage, the expressions for the coframe have simplified to

$$\begin{aligned} \alpha _1=\frac{1}{y}\beta +\gamma _1+O(y^1),\ \ \alpha _2=\frac{1}{y}{\textsf{d}}y+\gamma _2+O(y^1),\ \ \kappa =-\frac{1}{y}\beta +\gamma _3+O(y^1) \end{aligned}$$

(where we write $\beta =\beta _1$). From the sum of the first and third structure equations, ${\textsf{d}}(\alpha _1+\kappa )=-(\alpha _1+\kappa )\wedge \alpha _2$ we obtain, by comparing coefficients of $y^{-1}$, that $(\gamma _1+\gamma _3)\wedge {\textsf{d}}y=0$. On the other hand, ${\textsf{d}}\alpha _2=\kappa \wedge \alpha _1$ gives $(\gamma _1+\gamma _3)\wedge \beta =0$. Using again that $\beta ,{\textsf{d}}y$ are pointwise linearly independent, we conclude $\gamma _1+\gamma _3=0$, hence $\alpha _1+\kappa =O(y^1)$. $\square$

Locally, one can achieve an even better normal form for the coframe.

#### Lemma 3.7

Let $\textsf{g}$ be a hyperbolic 0-metric on $\Sigma$. For every $m\in \Sigma$ there exists an adapted oriented orthonormal coframe $\alpha _1,\alpha _2$ on some open neighborhood of *m* such that the associated spin connection is

$$\begin{aligned} \kappa =-\alpha _1. \end{aligned}$$

Equivalently, the connection 1-form (14) is upper triangular.

#### Proof

Begin by choosing any adapted oriented orthonormal coframe $\alpha _1,\alpha _2$ for $\textsf{g}$, and let *A* be the associated connection 1-form.

Consider the case that *m* is an interior point. Let $U\subseteq \Sigma$ be a simply connected open neighborhood of *m*. Since *A* is flat, it determines a parallel transport $g\in C^\infty (U,{\text {SL}}(2,\mathbb {R}))$, i.e. $A=g\bullet 0=-({\textsf{d}}g)g^{-1}$ with initial condition $g(m)=e$. The Iwasawa decomposition for ${\text {SL}}(2,\mathbb {R})$ gives a map $R(\psi ):U\rightarrow {\text {SO}}(2)$ such that $R(\psi ) g$ is upper triangular. It hence follows that $R(\psi )\bullet A=(R(\psi )g)\bullet 0$ is upper triangular.

The case that *m* is a boundary point requires more care, since we need a coframe rotation that extends all the way to the boundary. Pick adapted local coordinates *x*, *y* on a simply connected open neighborhood $U\subseteq \Sigma$ of *m*, and write $\alpha _1=\frac{1}{y} \beta +O(y^0)$ as in the proof of the previous lemma. Then

$$\begin{aligned} A= \left( \begin{array}{cc} \frac{1}{2y}\ {\textsf{d}}y+O(y^0) &  \frac{1}{y}\beta +O(y^0)\\ O(y^1) &  -\frac{1}{2y}\ {\textsf{d}}y+O(y^0)\end{array}\right) =\left( \begin{array}{cc} y^{-1/2}&  0\\ 0 &  y^{1/2}\end{array}\right) \bullet A',\end{aligned}$$

where $A'\in \Omega ^1(U,\mathfrak {g})$ is a *regular* flat connection on *U* – all matrix entries extend smoothly to the boundary. This determines a parallel transport $g':U\rightarrow {\text {SL}}(2,\mathbb {R})$ i.e. $A'=g'\bullet 0=-({\textsf{d}}g')g'^{-1}$ with initial condition $g'|_m=e$. Away from the boundary, we obtain $A=g\bullet 0$ with

19 $$\begin{aligned} g= \left( \begin{array}{cc} y^{-1/2}&  0\\ 0 &  y^{1/2}\end{array}\right) g':\ U-\partial \Sigma \rightarrow {\text {SL}}(2,\mathbb {R}). \end{aligned}$$

By the Iwasawa decomposition for ${\text {SL}}(2,\mathbb {R})$ there is a unique map $R(\psi ):U-\partial \Sigma \rightarrow {\text {SO}}(2)$ with the property that $R(\psi ) g$ is upper triangular with positive diagonal. With this choice, $R(\psi )\bullet A$ is upper triangular on $U-\partial \Sigma$. To show that $\psi$ extends smoothly to the boundary, write

20 $$\begin{aligned} g=\left( \begin{array}{cc} a&  b\\ c &  d\end{array}\right) ,\ \ g'= \left( \begin{array}{cc} a'&  b'\\ c' &  d'\end{array}\right) . \end{aligned}$$

Then

$$\begin{aligned} \psi =-\arctan (c/a)=-\arctan (y c'/a'). \end{aligned}$$

Since $a'|_m=1$, this extends smoothly to all of *U*. $\square$

#### Remark 3.8

The parallel transport (19), as a map into $G={\text {PSL}}(2,\mathbb {R})$, is well-defined only away from the boundary. It becomes well-defined up to the boundary if it is regarded as a map to the ‘wonderful’ compactification $\overline{G}$.

### Local normal form

Lemma 3.7 allows us to give a quick proof of the local normal form for hyperbolic 0-metrics. Earlier proofs proceed through uniformization and the classification of ends [6, Theorem 2.3], or through estimates for sectional curvatures [15]. (We thank D. Borthwick for these references.)

#### Theorem 3.9

Let $\textsf{g}$ be a hyperbolic 0-metric on $\Sigma$. Every $m\in \Sigma$ admits an open neighborhood *U* and a 0-isometry $U\rightarrow \overline{\mathbb {H}}$.

#### Proof

Consider the case that *m* is a boundary point. (For interior points the argument is similar.) By Lemma 3.7, we may choose an adapted oriented orthonormal coframe $\alpha _1,\alpha _2$ with $\kappa =-\alpha _1$. The second structure equation (13) gives ${\textsf{d}}\alpha _2=-\kappa \wedge \alpha _1=0$. Since the coframe is adapted, the difference $\alpha _2-\frac{{\textsf{d}}\varrho }{\varrho }$ extends smoothly to the boundary, and hence may be written as ${\textsf{d}}f$ near *m*. Hence, taking $y=e^f \varrho$ as a coordinate near *m*, we obtain $\alpha _2=y^{-1}{\textsf{d}}y$. The first structure equation shows that $y\alpha _1$ is closed: ${\textsf{d}}(y\alpha _1)={\textsf{d}}y\wedge \alpha _1-y\kappa \wedge \alpha _2=0$. We may therefore choose the coordinate *x* near *m* so that $y\alpha _1={\textsf{d}}x$, which gives $\alpha _1=y^{-1}\ {\textsf{d}}x$. This proves the existence of an isometric diffeomorphism from an open neighborhood *U* of $m\in \Sigma$ onto an open subset of $\bar{\mathbb {H}}$. $\square$

## Hyperbolic Structures from Flat Connections

The symplectic structure on the infinite-dimensional Teichmüller space ${\text {Teich}}(\Sigma )$ will be obtained by a reduction procedure, starting from the usual Atiyah–Bott symplectic structure on a space of connections. We encountered flat connection 1-forms in the coframe formalism, see (14). Note however that these 1-forms become singular at the boundary. While it is possible to work with these singular connections, we will pursue a different approach where (14) represents an *ordinary* connection 1-form $\theta$ on a principal bundle. This is motivated by the theory of geometric structures (cf. [12, 14, 34]).

Throughout, we take $G={\text {PSL}}(2,\mathbb {R})$, with the action on $\overline{\mathbb {D}}$ regarded as $\overline{\mathbb {D}}=\overline{\mathbb {H}}\cup \{\infty \}$, or equivalently via ${\text {PSL}}(2,\mathbb {R})\cong {\text {PSU}}(1,1)$ (cf. (11)).

### Flat bundles from hyperbolic structures

In Definition 2.1, hyperbolic structures on surfaces $\Sigma$ with boundary were described in terms of charts $\phi _\alpha :U_\alpha \rightarrow \overline{\mathbb {D}}$ with transition functions $h_{\alpha \beta }\in G$, i.e. $\phi _\alpha (x)=h_{\alpha \beta }. \phi _\beta (x)$ on $U_\alpha \cap U_\beta$. The transition functions define a principal *G*-bundle

$$\begin{aligned} \pi :P\rightarrow \Sigma , \end{aligned}$$

obtained from $\bigsqcup _\alpha (U_\alpha \times G)$ by identifying $(x,g)\in U_\beta \times G$ with $(x,h_{\alpha \beta }\,g)\in U_\alpha \times G$. The charts themselves determine a *G*-equivariant morphism of manifolds with boundary^1^

$$\begin{aligned} \sigma :P\rightarrow \overline{\mathbb {D}} \end{aligned}$$

given in the local trivializations by $U_\alpha \times G\rightarrow \overline{\mathbb {D}},\ (x,g)\mapsto g^{-1}. \phi _\alpha (x)$. We refer to $\sigma$ as a *developing section*, since it may be regarded as a section of the associated bundle with fiber $\overline{\mathbb {D}}$.

The principal bundle *P* comes equipped with a flat connection $\theta \in \mathcal {A}_{{\text {flat}}}(P)$, given in the defining local trivializations by $A_\alpha =0$. It has the following special property: Let ${\text {At}}(P)=TP/G$ be the Atiyah algebroid (see “Appendix B.1”.), and denote by $j^\theta :T\Sigma \rightarrow {\text {At}} (P)$ the horizontal lift defined by $\theta$. Then the composition

21 $$\begin{aligned} T\Sigma {\mathop {\longrightarrow }\limits ^{j^\theta }} {\text {At}} (P){\mathop {\longrightarrow }\limits ^{T\sigma }} V_\sigma =(\sigma ^*T\overline{\mathbb {D}})/G \end{aligned}$$

is an *orientation preserving bundle isomorphism*. In terms of the local trivialization $P|_{U_\alpha }=U_\alpha \times G$, we have $V_\sigma |_{U_\alpha }=\phi _\alpha ^*T\overline{\mathbb {D}}$, and (21) is just the tangent map $T\phi _\alpha :TU_\alpha \rightarrow V_\sigma |_{U_\alpha }=\phi _\alpha ^*T\overline{\mathbb {D}}$.

#### Remark 4.1

If the surface is the closed Poincaré disk itself, these constructions become tautological: The principal bundle is the trivial bundle $\overline{\mathbb {D}}\times G$ with the trivial connection ${\text {pr}}_2^*\theta ^L$ (where $\theta ^L$ is the left-invariant Maurer–Cartan form), and $\sigma (z,g)=g^{-1}. z$. All of these data are equivariant for the *G*-action on $\overline{\mathbb {D}}$. More generally, if $\Sigma =(\overline{\mathbb {D}}-\mathfrak {L})/\Gamma$ as in Remark 2.2, the triple $(P,\sigma ,\theta )$ for $\Sigma$ is obtained from the corresponding triple for $\overline{\mathbb {D}}-\mathfrak {L}$, by taking the quotient under $\Gamma$.

Similarly, any projective structure on an oriented 1-manifold $\textsf{C}$ (Definition 2.3) determines a principal *G*-bundle $Q\rightarrow \textsf{C}$ with a developing section $\tau :Q\rightarrow \partial \mathbb {D}$ and a connection $\vartheta \in \Omega ^1(Q,\mathfrak {g})$ such that the composition of maps

22 $$\begin{aligned} T\textsf{C}{\mathop {\longrightarrow }\limits ^{j^\vartheta }} {\text {At}} (Q){\mathop {\longrightarrow }\limits ^{T\tau }} V_\tau =(\tau ^*T\partial \mathbb {D})/G \end{aligned}$$

is an orientation preserving isomorphism. If $\textsf{C}=\partial \Sigma$, and the projective structure on $\textsf{C}$ is induced by a hyperbolic structure on $\Sigma$, then $Q=\partial P$ is the restriction of *P*, with $\tau =\partial \sigma$ the restriction of $\sigma$, and with connection 1-form $\vartheta =\partial \theta$ the pullback of $\theta$.

### Hyperbolic structures from flat bundles

We shall now reverse the procedure and take as our starting point the data23of a principal *G*-bundle and a *developing section* $\sigma$ (a *G*-equivariant morphism of manifolds with boundary). Over the interior ${\text {int}}(\Sigma )$, the map $\sigma$ takes values in $\mathbb {D}=G/K$ (where $K\cong {\text {SO}}(2)$ is the stabilizer of $i\in \mathbb {H}\cong \mathbb {D}$), and so defines a reduction of structure group

24 $$\begin{aligned} P_K\subseteq P|_{{\text {int}}(\Sigma )}. \end{aligned}$$

On the other hand, the boundary restriction $\partial \sigma :\partial P=P|_{\partial \Sigma }\rightarrow \partial \mathbb {D}$ takes values in $\partial \mathbb {D}=G/B^-$, where $B^-$ is the stabilizer of $0\in \partial \mathbb {H}\cup \{\infty \} \cong \partial \mathbb {D}$, and so defines a reduction of structure group

25 $$\begin{aligned} (\partial P)_{B^-}\subseteq \partial P. \end{aligned}$$

Note that $B^-\subseteq G={\text {PSL}}(2,\mathbb {R})$ is the image of the group of lower triangular matrices with positive diagonal entries. It is isomorphic to $\mathbb {R}\rtimes \mathbb {R}_{>0}$; in particular it is *contractible*. The role of $\sigma$ is to combine these two reductions of structure group: to *K* over the interior, and to $B^-$ over the boundary.

#### Definition 4.2

A connection $\theta \in \mathcal {A}(P)$ is called $\sigma$-*positive* (or simply *positive*, if $\sigma$ is understood) if the map $T\Sigma \rightarrow V_\sigma$ given in (21) is an orientation preserving isomorphism.

Denote by $\mathcal {A}^{{\text {pos}}}(P)$ the space of positive connections, and by $\mathcal {A}^{{\text {pos}}}_{{\text {flat}}}(P)$ those which are furthermore flat. There is a natural map from $\mathcal {A}^{{\text {pos}}}(P)$ to the space of 0-metrics: The standard 0-metric on $T\overline{\mathbb {D}}$ gives a 0-metric on $V_\sigma =\sigma ^* T\overline{\mathbb {D}}/G$; a positive connection gives an isomorphism $T\Sigma \cong V_\sigma$.

#### Proposition 4.3

If $\theta \in \mathcal {A}^{{\text {pos}}}(P)$ is flat, then the 0-metric $\textsf{g}$ defined by $\theta$ is hyperbolic.

#### Proof

Choose local trivializations $P|_{U_\alpha }\cong U_\alpha \times G$ taking $\theta$ to the trivial connection, and write $\sigma (x,g)=g^{-1}. \phi _\alpha (x)$ in terms of the trivialization. The positivity condition ensures that the maps $\phi _\alpha :U_\alpha \rightarrow \overline{\mathbb {D}}$ are orientation preserving diffeomorphisms onto their image, and so define a hyperbolic structure. Clearly, $\textsf{g}$ is the 0-metric associated to this hyperbolic structure. $\square$

#### Remark 4.4

The construction may also be understood as follows: A flat connection $\theta$ determines a horizontal foliation of *P*. Positivity means exactly that $\sigma$ restricts to orientation-preserving local diffeomorphisms from the horizontal leaves to $\overline{\mathbb {D}}$. Hence, the hyperbolic structure on $\overline{\mathbb {D}}$ pulls back to a *G*-invariant hyperbolic structure on the horizontal foliation, which then descends to $\Sigma$.

Given an oriented 1-manifold $\textsf{C}$, we may similarly consider the data26of a principal *G*-bundle over $\textsf{C}$ with a developing section (a *G*-equivariant map to $\partial \mathbb {D}\cong \mathbb {R}\!{\text {P}}(1)$). A connection $\vartheta$ on *Q* is called positive if the map (22) is an orientation preserving isomorphism. Such a connection defines a projective structure on $\textsf{C}$; conversely, every projective structure on $\textsf{C}$ arises in this way.

Returning to the pair $(P,\sigma )$ for surfaces with boundary, we have:

#### Proposition 4.5

A connection $\theta \in \mathcal {A}(P)$ satisfies the $\sigma$-positivity condition along the boundary $\partial {\Sigma }$ if and only if the pullback connection $\partial \theta \in \mathcal {A}(\partial P)$ is $\partial \sigma$-positive.

#### Proof

The map $T\Sigma \rightarrow V_\sigma$ given by $\theta$ restricts to the map $T\partial {\Sigma }\rightarrow V_{\partial \sigma }$ given by $\partial \theta$. The resulting map on quotients,

$$\begin{aligned} \nu (\Sigma ,\partial {\Sigma })\rightarrow V_\sigma |_{\partial \Sigma }/V_{\partial \sigma },\end{aligned}$$

does not depend on the choice of $\theta$. In fact, it is simply the map obtained by applying the normal bundle functor to the map of pairs $\sigma :(P,\partial P)\rightarrow (\mathbb {D},\partial \mathbb {D})$, using that

$$\begin{aligned} \nu (\Sigma ,\partial \Sigma )=\nu (P,\partial P)/G,\ \ \ \ V_\sigma |_{\partial \Sigma }/V_{\partial \sigma }= (\partial \sigma )^*\nu (\mathbb {D},\partial \mathbb {D})/G. \end{aligned}$$

In particular, the map on quotients is always an orientation preserving isomorphism. We conclude that $T\Sigma |_{\partial {\Sigma }}\rightarrow V_\sigma |_{\partial {\Sigma }}$ is an orientation preserving isomorphism if and only if $T\partial {\Sigma }\rightarrow V_{\partial \sigma }$ is an orientation preserving isomorphism. $\square$

### Relationship with coframe formalism

Given $(P,\sigma )$, consider the reduction of structure group (24) to $K\subseteq G$. A trivialization of $P_K$ over $U\subseteq {\text {int}}(\Sigma )$ determines a trivialization $P|_U=U\times G$ such that $\sigma (m,g)=g^{-1}. i$. Let $A\in \Omega ^1(U,\mathfrak {g})$ be the connection 1-form of $\theta$ in this trivialization. Define 1-forms $\alpha _1,\alpha _2$, and $\kappa$ by writing

27 $$\begin{aligned} A={\frac{1}{2}}\left( \begin{array}{cc}\alpha _2& \alpha _1-\kappa \\ \alpha _1+\kappa & -\alpha _2 \end{array} \right) . \end{aligned}$$

#### Proposition 4.6

The connection $\theta \in \mathcal {A}(P)$ is positive over *U* if and only if $\alpha _1,\alpha _2$ are an oriented coframe. In this case, $\alpha _1,\alpha _2$ is an orthonormal coframe for the metric $\textsf{g}$ defined by $\theta$; if the connection is flat then $\kappa$ is the spin connection for this coframe.

#### Proof

Since $T\mathbb {D}\cong T\mathbb {H}=T(G/K)=G\times _K \mathfrak {k}^\perp$, we have

$$\begin{aligned} V_\sigma |_{{\text {int}}(\Sigma )}=(P_K\times \mathfrak {k}^\perp )/K=\mathfrak {k}^\perp (P_K). \end{aligned}$$

Hence, $V_\sigma |_U=U\times \mathfrak {k}^\perp$, and (21) becomes a map

28 $$\begin{aligned} TU\rightarrow U\times \mathfrak {k}^\perp . \end{aligned}$$

Viewed as an element of $\Omega ^1(U,\mathfrak {k}^\perp )$, this map is the symmetric part of the connection 1-form *A*. The condition that (28) is an orientation preserving isomorphism means exactly that $\alpha _1,\alpha _2$ is an oriented orthonormal coframe. Finally, the metric on $V_\sigma |_U$ corresponds to the standard metric on $\mathfrak {k}^\perp =T_i\mathbb {H}$, and the metric on *TU* induced by (28) is exactly the one defined by the coframe $\alpha _1,\alpha _2$. $\square$

The reduction of structure group to *K* does not extend to the boundary. To describe the limiting behaviour, we shall work with the following lemma. Let $\varrho \in C^\infty (\Sigma )$ be a boundary defining function.

#### Lemma 4.7

(Normal form at boundary) Given $(P,\sigma )$, there exists $\epsilon >0$ and a trivialization $P|_U=U\times G$ over $U=\varrho ^{-1}\big ([0,\epsilon )\big )$ such that, in terms of the trivialization,

$$\begin{aligned} \sigma (m,g)=g^{-1}. (i\varrho (m)). \end{aligned}$$

As usual, we identify $\overline{\mathbb {D}}\cong \overline{\mathbb {H}}\cup \{\infty \}$; thus $i\varrho (m)$ is regarded as an element of the closed upper half plane.

#### Proof

The choice of a trivialization of $\sigma ^{-1}(0)=(\partial P)_{B^-}$ gives a trivialization $\partial P=\partial {\Sigma }\times G$ such that $(\partial \sigma )(m,g)=g^{-1}. 0$ for $m\in \partial {\Sigma }$. Extend it to a trivialization of *P* over $U=\varrho ^{-1}\big ([0,\epsilon )\big )$ for some $\epsilon >0$. In terms of this trivialization, $\sigma$ is of the form

$$\begin{aligned} \sigma (m,g)=g^{-1}. f(m),\end{aligned}$$

where $f:U\rightarrow \overline{\mathbb {D}}\cong \overline{\mathbb {H}}\cup \{\infty \}$ is a morphism of manifolds with boundary, with $f|_{\partial {\Sigma }}=0$. Taking $\epsilon$ smaller if needed, we may assume *f* takes values in $\overline{\mathbb {H}}\subseteq \overline{\mathbb {D}}$. In particular, the imaginary part ${\text {Im}}(f)$ is a boundary defining function, and so $\varrho =u^2\, {\text {Im}}(f)$ for some function $u\in C^\infty (U,\mathbb {R}_{>0})$. We have

$$\begin{aligned} \left[ \begin{array}{cc} u&  0\\ 0 &  u^{-1} \end{array}\right] \left[ \begin{array}{cc} 1&  -{\text {Re}}(f)\\ 0 &  1 \end{array}\right] . f= \left[ \begin{array}{cc} u&  0\\ 0 &  u^{-1} \end{array}\right] . i{\text {Im}}(f) = i\varrho \end{aligned}$$

Hence, a gauge transformation by $$\left[ \begin{array}{cc} u&  0\\ 0 &  u^{-1} \end{array}\right] \left[ \begin{array}{cc} 1&  -{\text {Re}}(f)\\ 0 &  1 \end{array}\right] $$ replaces $\sigma (m,g)=g^{-1}. f(m)$ with $g^{-1}. i\varrho (m)$. $\square$

Given a connection $\theta \in \mathcal {A}(P)$, let $A\in \Omega ^1(U,\mathfrak {g})$ be its connection 1-form in terms of the trivialization from this lemma. Over $U-\partial \Sigma$, the gauge transformation by

29 $$\begin{aligned} h=\left[ \begin{array}{cc}\rho ^{1/2}&  0 \\ 0 &  \rho ^{-1/2}\end{array}\right] :U-\partial {\Sigma }\rightarrow G \end{aligned}$$

is defined, and satisfies $h^{-1}. i\varrho (m)=i$. Hence, $(h^{-1}. \sigma )(m,g)=g^{-1}. i$, and $h^{-1}\bullet A$ is of the form (27), defining $\alpha _1,\alpha _2,\kappa$. As we saw above, the positivity condition on $\theta$ means that over the interior, $\alpha _1,\alpha _2$ are an oriented orthonormal coframe. The original connection 1-form is expressed in terms of these data as

$$\begin{aligned} A={\frac{1}{2}}\left( \begin{array}{cc}\alpha _2-\varrho ^{-1}{\textsf{d}}\varrho & \varrho (\alpha _1-\kappa )\\ \varrho ^{-1}(\alpha _1+\kappa )& -\alpha _2 +\varrho ^{-1}{\textsf{d}}\varrho \end{array} \right) .\end{aligned}$$

Since *A* is a *regular* connection 1-form, this shows that $\alpha _1,\alpha _2$ extend to elements of $^0\Omega ^1(U)$, and define an adapted orthonormal coframe.

### Existence of positive connections

Recall the classification of principal bundles over compact, connected, oriented surfaces $\Sigma$ with boundary. Let *H* be a connected Lie group, and $R\rightarrow \Sigma$ a principal *H*-bundle with a given homotopy class of trivializations (framings) of $R|_{\partial {\Sigma }}$. Pick $x_0\in {\text {int}}(\Sigma )$, and choose a trivialization of *R* over the punctured surface $\Sigma -\{x_0\}$ such that the trivialization along the boundary is in the prescribed class. Also choose a trivialization of *R* over an embedded disk $D\subseteq {\text {int}}(\Sigma )$ around $x_0$. The homotopy class of the transition map $D-\{x_0\}\rightarrow H$ defines an element

$$\begin{aligned} \textsf{e}(R)\in \pi _1(H). \end{aligned}$$

If $R_1,R_2$ are two principal *H*-bundles with homotopy classes of trivializations along the boundary, we have $\textsf{e}(R_1)=\textsf{e}(R_2)$ if and only if there exists a bundle isomorphism $R_1\rightarrow R_2$ which intertwines the homotopy classes of trivializations over the boundary. In particular, taking $R={\text {Fr}}_{ {\text {SO}}(2)}(\Sigma )$ to be the oriented orthonormal frame bundle for a Riemannian metric, with its standard trivialization along the boundary (where the first element of a frame is tangent to the boundary, pointing in the positive direction), the element $\textsf{e}(R)\in \pi _1( {\text {SO}}(2))=\mathbb {Z}$ is the Euler characteristic $\chi (\Sigma )$ of the surface. Given a principal *G*-bundle $P\rightarrow \Sigma$ with developing section $\sigma :P\rightarrow \overline{\mathbb {D}}=\overline{\mathbb {H}}\cup \{\infty \}$ as above, there is a distinguished homotopy class of trivializations along the boundary – those trivializations for which $\sigma (m,g)=g^{-1}. 0$ for $m\in \partial {\Sigma }$. Let

$$\begin{aligned} \textsf{e}(P,\sigma )\in \pi _1(G)=\mathbb {Z}\end{aligned}$$

be the resulting invariant.

#### Proposition 4.8

Given two pairs $(P,\sigma )$ and $(P',\sigma ')$, we have $\textsf{e}(P,\sigma )=\textsf{e}(P',\sigma ')$ if and only if there exists an isomorphism $P\rightarrow P'$ taking $\sigma$ to $\sigma '$ and inducing the identity on the base.

#### Proof

The necessity of the condition is obvious. To show that it is also sufficient, suppose $\textsf{e}(P,\sigma )=\textsf{e}(P',\sigma ')$. We may assume $P=P'$, and that $\sigma ,\sigma '$ define the same homotopy class of trivializations of $\partial P=P|_{\partial {\Sigma }}$. Using the normal form near the boundary (Lemma 4.7), we may assume that $\sigma ,\sigma '$ coincide over an open neighborhood of the boundary. Over the interior, $\sigma ,\sigma '$ may be regarded as sections of the associated bundle with fiber $\mathbb {D}$, which agree near the boundary. As is well-known, given any two distinct points $z,z'\in \mathbb {D}$ there is a unique element $\phi (z,z')\in G$ taking *z* to $z'$ and preserving the geodesic through $z,z'$. It extents smoothly to a map $\phi :\mathbb {D}\times \mathbb {D}\rightarrow G$ with $\phi (g.z,g.z')=g\phi (z,z') g^{-1}$. Consequently,we obtain a gauge transformation $h\in {\text {Gau}} (P)$ taking $\sigma$ to $\sigma '$. This gauge transformation is trivial near the boundary since $\sigma ,\sigma '$ agree there. $\square$

The existence of $\sigma$-positive connections places a topological condition on $(P,\sigma )$.

#### Proposition 4.9

Let $\Sigma$ be a compact, connected, oriented surface with boundary, and $P\rightarrow \Sigma$ a principal *G*-bundle with a developing section $\sigma :P\rightarrow \overline{\mathbb {D}}$. Then the space of positive connections is empty unless $\textsf{e}(P,\sigma )=\chi (\Sigma )$.

#### Proof

The oriented rank 2 bundle $V_\sigma |_{\partial \Sigma }$ has a distinguished rank 1 subbundle $V_{\partial \sigma }$; hence there is a unique homotopy class of trivializations of $V_\sigma$ along the boundary, taking the subbundle to $\mathbb {R}\oplus 0\subseteq \mathbb {R}^2$. Letting ${\text {Fr}}_{ {\text {SO}}(2)}(V_\sigma )$ be the frame bundle for (any) fiber metric, the invariant $\textsf{e}({\text {Fr}}_{ {\text {SO}}(2)}(V_\sigma ))\in \pi _1( {\text {SO}}(2))=\mathbb {Z}$ is defined. We claim that

30 $$\begin{aligned} \textsf{e}({\text {Fr}}_{ {\text {SO}}(2)}(V_\sigma ))=\textsf{e}(P,\sigma ). \end{aligned}$$

To see this, choose a covering of $\Sigma$, consisting of an open subset $U_1=\Sigma -\{x_0\}$ where $x_0\in {\text {int}}(\Sigma )$, and an open neighborhood $U_2$ of $x_0$, contained in the interior of $\Sigma$ and diffeomorphic to an open disk. Choose a trivialization of *P* over $U_1$, inducing the given class of trivializations along the boundary, and choose also a trivialization over $U_2$. Let $f_i:U_i\rightarrow \overline{\mathbb {D}}$ be the maps describing $\sigma$ in these trivializations. We may arrange that $f_1,f_2$ are both constant (equal to *i*) over $U_1\cap U_2$. Then the transition map $U_2-\{x_0\}=U_1\cap U_2\rightarrow G$ takes values in *K*. The trivializations of $P|_{U_i}$ also trivialize $V_\sigma |_{U_i}$, and the transition map for its frame bundle agrees with that for *P* under the isomorphism $K\cong {\text {SO}}(2)$. This proves (30).

A positive connection determines an oriented vector bundle isomorphism $T\Sigma \rightarrow V_\sigma$, and hence

$$\begin{aligned} \chi (\Sigma )=\textsf{e}({\text {Fr}}_{ {\text {SO}}(2)}(T\Sigma ))=\textsf{e}({\text {Fr}}_{ {\text {SO}}(2)}(V_\sigma ))=\textsf{e}(P,\sigma ). \end{aligned}$$

$$\square $$

## Automorphisms

In this section, we discuss the structure of the groups of automorphisms preserving a given developing section, for *G*-bundles over surfaces and over curves. Recall that $B^-\subseteq G={\text {PSL}}(2,\mathbb {R})$ is the image of lower triangular matrices with positive diagonal entries. Thus $B^-=AN^-$ where *A* is the image of positive diagonal matrices, and $N^-=[B^-,B^-]$ is the image of lower triangular matrices with 1’s on the diagonal.

### The group ${\text {Aut}} (Q,\tau )$

Let $Q\rightarrow \textsf{C}$ be a principal *G*-bundle over a compact oriented 1-manifold, and $\tau :Q\rightarrow \partial \mathbb {D}=\partial \mathbb {H}\cup \{\infty \}$ a developing section.

#### Proposition 5.1

The group ${\text {Gau}} (Q,\tau )$ of gauge transformations preserving $\tau$ is the group of sections of a group bundle $G(Q,\tau )\rightarrow \textsf{C}$, with typical fiber $B^-=AN^-$. It fits into an exact sequence with the group ${\text {Aut}} (Q,\tau )$ of automorphisms of preserving $\tau$,

$$\begin{aligned} 1\rightarrow {\text {Gau}} (Q,\tau )\rightarrow {\text {Aut}} (Q,\tau )\rightarrow {\text {Diff}}(\textsf{C})\rightarrow 1. \end{aligned}$$

Infinitesimally, $\mathfrak {gau} (Q,\tau )\subseteq \mathfrak {aut} (Q,\tau )$ are the sections of a Lie algebroids $\mathfrak {g}(Q,\tau )\subseteq {\text {At}} (Q,\tau )$, described as the kernel of the bundle maps $\mathfrak {g}(Q)\subseteq {\text {At}} (Q)\rightarrow V_\tau$. We have an exact sequence of Lie algebroids

$$\begin{aligned} 0\rightarrow \mathfrak {g}(Q,\tau )\rightarrow {\text {At}} (Q,\tau )\rightarrow T\textsf{C}\rightarrow 0. \end{aligned}$$

#### Proof

The developing section defines a reduction of structure group $Q_{B^-}=\tau ^{-1}(0)\subseteq Q$, and the groups ${\text {Gau}} (Q,\tau )\subseteq {\text {Aut}} (Q,\tau )$ are identified with the gauge transformations and automorphisms of $Q_{B^-}$. (Every $B^-$-equivariant diffeomorphism of $Q_{B^-}$ extends uniquely to a *G*-equivariant diffeomorphism of *Q*; the latter preserves $\tau$.) In particular, ${\text {At}} (Q,\tau )$ is just the Atiyah algebroid of $Q_{B^-}$. The sections of ${\text {At}} (Q,\tau )$ are identified with the $B^-$-invariant vector fields on $Q_{B^-}$, or equivalently with the *G*-invariant vector fields on *Q* that are $\tau$-related to 0. Equivalently, this is the kernel of the bundle map to $V_\tau$. $\square$

### The group ${\text {Aut}} (P,\sigma )$

We now give a similar discussion for principal bundles over oriented surfaces $\Sigma$ with boundary. Let$(P,\sigma )$ as in (23). As we saw, $\sigma$ gives a simultaneous description of two reductions of structure group: Over the interior, the structure group of *P* is reduced to *K*, over $\partial {\Sigma }$ it is reduced to $B^-$.

#### Proposition 5.2

The groups of gauge transformations and automorphism of *P* preserving $\sigma$ fit into an exact sequence

$$\begin{aligned} 1\rightarrow {\text {Gau}} (P,\sigma )\rightarrow  ^b {\text {Aut}} (P,\sigma )\rightarrow  ^b {\text {Diff}}(\Sigma )\rightarrow 1.\end{aligned}$$

The group ${\text {Gau}} (P,\sigma )$ is the group of sections of a family of Lie groups $G(P,\sigma )$, with typical fibers *K* over interior points and $N^-$ at boundary points; restriction to the boundary identifies

$$\begin{aligned} G(P,\sigma )|_{\partial {\Sigma }} =[G(\partial P,\partial \sigma ),G(\partial P,\partial \sigma )].\end{aligned}$$

(A *family of Lie groups* is a Lie groupoid for which source and target map coincide. It need not be locally trivial.)

#### Proof

Over the interior of $\Sigma$, the developing section defines a reduction of the structure group to $K\subseteq G$, and the groups ${\text {Gau}} (P|_{{\text {int}}(\Sigma )},\sigma )\subseteq {\text {Aut}} (P|_{{\text {int}}(\Sigma )},\sigma )$ are identified with the gauge transformations and automorphisms of $P_K$. The main task in proving Proposition 5.2 is to understand the behavior near the boundary. We shall use the local normal form, Lemma 4.7. Thus let

$$\begin{aligned}U=\partial \Sigma \times [0,\epsilon ),\ \ P|_U=U\times G,\end{aligned}$$

with $\sigma (m,g)=g^{-1}. (i\varrho (m))$. Denote the points of *U* by $m=(x,y)$, so that $\varrho (m)=y$. The automorphisms of $P|_U$ may be written as pairs $(h,\Phi )$, where $\Phi \in  ^b {\text {Diff}}(U)$ and $h\in {\text {Gau}} (P)=C^\infty (U,G)$. Such an automorphism preserves the developing section $\sigma (m,g)=g^{-1}.f(m)$ if and only if

$$\begin{aligned} h(m).f(\Phi ^{-1}(m))=f(m).\end{aligned}$$

In our case, $f(x,y)=iy$, we have:

#### Lemma 5.3

The elements of ${\text {Gau}} (P|_U,\sigma )$ with compact support in *U* are of the form

$$\begin{aligned}\exp \Big ( \begin{array}{cc} 0 &  -\chi \varrho ^2\\ \chi &  0 \end{array}\Big )\end{aligned}$$

for compactly supported $\chi \in C^\infty (U)$. Every element of $^b \! {\text {Aut}} (P|_U,\sigma )$ whose base map is compactly supported in *U* is uniquely the product of such a gauge transformation and an automorphism

$$\begin{aligned} \left( \Big [ \begin{array}{cc} e^{-\lambda /2}&  0\\ 0 &  e^{\lambda /2} \end{array}\Big ],\ \Phi \right) \end{aligned}$$

where $\Phi \in  ^b {\text {Diff}}(U)$ has compact support in *U*, and $\lambda \in C^\infty (U)$ is the compactly supported function defined by $\Phi _*\varrho =e^\lambda \,\varrho$.

#### Proof

A gauge transformation $h\in C^\infty (U,G)$ fixes $\sigma$ if and only if $h(x,y)\in G_{iy}$ for all *y*. For $y>0$, the stabilizer of $iy\in \mathbb {H}$ in *G* is

31 $$\begin{aligned} G_{iy}=\left\{ \exp \Big (\begin{array}{cc} 0 &  -t\,y^2\\ t &  0 \end{array}\Big )\Big |\ t\in \mathbb {R}\right\} \cong K\end{aligned}$$

This fits uniquely into a smooth family of subgroups of $\{(x,y)\}\times G$ for all $y\ge 0$, by taking the fiber for $y=0$ to be $N^-$. A smooth gauge transformation fixing $\sigma$ must take values in this family of Lie groups. Consider next a compactly supported diffeomorphism $\Phi \in  ^b {\text {Diff}}_+(U)$. The push-forward $\Phi _*\varrho =\varrho \circ \Phi ^{-1}$ is again a boundary defining function, and so is of the form $e^\lambda \varrho$. The hyperbolic transformation given by the diagonal matrix with entries $e^{-\lambda (m)/2},e^{\lambda (m)/2}$ down the diagonal takes $f(\Phi ^{-1}(m))=i\,e^{\lambda (m)} y$ back to *iy*.

The Lemma (and its proof) verify that ${\text {Gau}} (P,\sigma )$ are the sections of a family of Lie groups $G(P,\sigma )$. It also shows that every diffeomorphism in $^b {\text {Diff}}(\Sigma )$ lifts to an automorphism in $^b {\text {Aut}} (P,\sigma )\rightarrow  ^b {\text {Diff}}(\Sigma )$: For diffeomorphisms supported in the interior of $\Sigma$ this is done by lifting to an automorphisms of $P_K\subseteq P_{{\text {int}}(\Sigma )}$; for diffeomorphisms supported in a collar neighborhood of the boundary the Lemma gives an explicit lift.$\square$

We see in particular that the restriction map ${\text {Gau}} (P,\sigma )\rightarrow {\text {Gau}} (\partial P,\partial \sigma )$ is *not* surjective. On the other hand, we have:

#### Proposition 5.4

The restriction map

$$\begin{aligned}  ^b \!\! {\text {Aut}} _\textsf{o}(P,\sigma )\rightarrow {\text {Aut}} _\textsf{o}(\partial P,\partial \sigma )\end{aligned}$$

is surjective. In fact, every element of ${\text {Aut}} _\textsf{o}(\partial P,\partial \sigma )$ admits an extension to an element of $^b \! {\text {Aut}} _\textsf{o}(P,\sigma )$ which is supported on a collar neighborhood of the boundary.

#### Proof

We work with the normal form (Lemma 4.7) over a collar neighborhood *U* of the boundary. Given an element of ${\text {Aut}} _\textsf{o}(\partial P,\partial \sigma )$, with base map $\partial \Phi \in {\text {Diff}}_\textsf{o}(\partial \Sigma )$, choose an extension to a diffeomorphism $\Phi$ with compact support on *U*. Lemma 5.3 shows how to lift $\Phi$ to an element of $^b \! {\text {Aut}} _\textsf{o}(P,\sigma )$, extending the given automorphism along the boundary. $\square$

For the sake of completeness, we also give the infinitesimal descriptions. Recall that the *b*-tangent bundle $$ ^b TM$$ of a manifold *M* with boundary is the vector bundle whose sections are vector fields tangent to the boundary $$\partial M$$.

#### Proposition 5.5

The kernel of bundle map ${\text {At}} (P)\rightarrow V_\sigma$ is a Lie subalgebroid $^b {\text {At}} (P,\sigma )$ of the Atiyah algebroid, with $^b \mathfrak {aut} (P,\sigma )$ as its space of sections. It fits into an exact sequence of Lie algebroids

32 $$\begin{aligned} 0\longrightarrow \mathfrak {g}(P,\sigma )\longrightarrow {\text {At}} (P,\sigma )\longrightarrow  ^b T\Sigma \longrightarrow 0.\end{aligned}$$

Restriction to the boundary is a Lie algebroid isomorphism

33 $$\begin{aligned} {\text {At}} (P,\sigma )|_{\partial \Sigma }\cong {\text {At}} (\partial P,\partial \sigma ) \end{aligned}$$

inducing the inclusion $\mathfrak {g}(P,\sigma )|_{\partial {\Sigma }}\hookrightarrow \mathfrak {g}(\partial P,\partial \sigma )$.

#### Proof

Over the the interior, the kernel of ${\text {At}} (P)\rightarrow V_\sigma$ is the subalgebroid ${\text {At}} (P_K)$ given by the reduction of structure group $P_K\subseteq P|_{{\text {int}}(\Sigma )}$. Hence, it suffices to study the situation near the boundary. Using the normal form from Lemma 4.7, we have $P|_U=U\times G$, with $\sigma (m,g)=g^{-1}.f(m)$ for $f(x,y)=iy$. The bundle map ${\text {At}} (P|_U)=TU\times \mathfrak {g}\rightarrow V_\sigma |_U=f^*T\overline{\mathbb {H}}$ is given by

$$\begin{aligned} (v,\xi )\mapsto (T_mf)(v)-\xi ^\sharp |_{f(m)},\end{aligned}$$

where $\xi ^\sharp$ is the vector field on $\overline{\mathbb {H}}$ generated by *X*. Since $\xi ^\sharp$ is tangent to $\partial \mathbb {H}$, we see that for elements $(v,\xi )\in {\text {At}} (P)|_m,\ m\in \partial {\Sigma }$ in the kernel of the map to $V_\sigma$, the vector *v* must itself be tangent to $\partial \Sigma$. The rest of the discussion is as in the proof of Lemma 5.3. In particular, infinitesimal gauge transformations are given by functions

$$\begin{aligned} \chi \left( \begin{array}{cc} 0 &  -\varrho ^2\\ 1 &  0 \end{array}\right) \in C^\infty (U,\mathfrak {g})= \mathfrak {gau} (P|_U).\end{aligned}$$

Every compactly supported vector field $v\in  ^b {\text {Vect}}(U)$ tangent to $\partial \Sigma$ defines a function $\lambda =\varrho ^{-1}\mathcal {L}_v \varrho$, and the element

$$\begin{aligned} \left( \Big ( \begin{array}{cc} -\lambda /2&  0\\ 0 &  \lambda /2 \end{array}\Big ),\ v\right) \in C^\infty (U,\mathfrak {g})\times {\text {Vect}}(U)= \mathfrak {aut} (P|_U) \end{aligned}$$

is a lift to $\mathfrak {aut} (P|_U,\sigma )$. $\square$

#### Remark 5.6

For the model case $\Sigma =\overline{\mathbb {D}}$,

the Lie algebroid ${\text {At}} (P,\sigma )$ is identified with the action Lie algebroid $\overline{\mathbb {D}}\times \mathfrak {g}$, embedded in ${\text {At}} (P)=T\overline{\mathbb {D}}\times \mathfrak {g}$ by the map $(z,\xi )\mapsto (\xi ^\sharp (z),\xi )$.

## Symplectic Structure on ${\text {Teich}}(\Sigma )$

We shall now construct a symplectic structure $\omega$ on the Teichmüller spaces of hyperbolic structures on surfaces $\Sigma$ with boundary. Throughout, $\Sigma$ will be compact, connected, and oriented, with a given pair $(P,\sigma )$ as in (23), satisfying $\textsf{e}(P,\sigma )=\chi (\Sigma )$. By Proposition 4.9, the space $\mathcal {A}^{{\text {pos}}}(P)$ of $\sigma$-positive connections is non-empty.

### Hyperbolic metrics from positive connections

Our starting point is the following description of ${\text {Hyp}}(\Sigma ),\ {\text {Teich}}(\Sigma )$ as quotients of spaces of flat connections.

#### Theorem 6.1

The space of hyperbolic structures on $\Sigma$ is a quotient,

34 $$\begin{aligned} {\text {Hyp}}(\Sigma )=\mathcal {A}_{{\text {flat}}}^{{\text {pos}}}(P)/ {\text {Gau}} (P,\sigma ). \end{aligned}$$

Taking a further quotient by the action of $^0{\text {Diff}}_\textsf{o}(\Sigma )$, we obtain

35 $$\begin{aligned} {\text {Teich}}(\Sigma )&=\mathcal {A}_{{\text {flat}}}^{{\text {pos}}}(P)/ ^0\!\! {\text {Aut}} _\textsf{o}(P,\sigma ). \end{aligned}$$

#### Proof

By Proposition 4.3, every $\theta \in \mathcal {A}^{{\text {pos}}}_{{\text {flat}}}(P)$ determines a hyperbolic 0-metric $\textsf{g}$ on $\Sigma$; changing $\theta$ by a gauge transformation in ${\text {Gau}} (P,\sigma )$ does not change $\textsf{g}$. Conversely, every hyperbolic 0-metric on $\Sigma$ arises in this way, where $\theta$ is unique up to the action of ${\text {Gau}} (P,\sigma )$. Indeed, $\textsf{g}$ determines a triple $(P',\sigma ',\theta )$, with $\textsf{e}(P',\sigma ')=\chi (\Sigma )$; hence $(P',\sigma ')$ is related to $(P,\sigma )$ by a bundle isomorphism. It follows that $\textsf{g}$ is defined by a flat connection $\theta \in \mathcal {A}^{{\text {pos}}}_{{\text {flat}}}(P)$ (the image of $\theta '$ under this isomorphism). This proves the description of ${\text {Hyp}}(\Sigma )$; the remaining assertions are clear. $\square$

Similarly, given $(Q,\tau )$ as in (26), we saw that every projective structure on an oriented 1-manifold $\textsf{C}$ is obtained from a $\tau$-positive connection on *Q*. The latter is unique up to the action of ${\text {Gau}} (Q,\tau )$. Hence

$$\begin{aligned} {\text {Proj}}(\textsf{C})=\mathcal {A}^{{\text {pos}}}(Q)/ {\text {Gau}} (Q,\tau ).\end{aligned}$$

The quotient map intertwines the action of ${\text {Aut}} _+(Q,\tau )$ on connections with the action of ${\text {Diff}}_+(\textsf{C})= {\text {Aut}} _+(Q,\tau )/ {\text {Gau}} (Q,\tau )$ on projective structures. Letting $Q=\partial P$ and $\tau =\partial \sigma$, we see that the pullback map $\mathcal {A}^{{\text {pos}}}_{{\text {flat}}}(P)\rightarrow \mathcal {A}^{{\text {pos}}}(\partial P)$ descends to the natural map ${\text {Teich}}(\Sigma )\rightarrow {\text {Proj}}(\partial {\Sigma })$.

### Atiyah–Bott symplectic structure

Let $\cdot$ denote the nondegenerate invariant symmetric bilinear form (‘metric’) on $\mathfrak {g}=\mathfrak {sl}(2,\mathbb {R})$ given by

36 $$\begin{aligned} \xi \cdot \eta ={\text {tr}}(\xi \eta ). \end{aligned}$$

It determines a bundle metric on $\mathfrak {g}(P)$, and an Atiyah–Bott symplectic structure on the space $\mathcal {A}(P)$ of connections by

$$\begin{aligned} \omega _{AB}(a,b)=\int _\Sigma a{\mathop {\wedge }\limits ^{.}} b, \end{aligned}$$

for $a,b\in T_\theta \mathcal {A}(P)=\Omega ^1(\Sigma ,\mathfrak {g}(P))$. The symplectic structure is invariant under the action of $^b \! {\text {Aut}} _+(P)$, and there is a natural affine moment map for this action, involving both a bulk term and a boundary term. We refer to “Appendix B” for details.

For now, we consider the subgroup $^0\! {\text {Aut}} _+(P)$ of automorphisms whose base map fixes the boundary. The moment map for this subgroup is given by

37 $$\begin{aligned} \mathcal {A}(P)\rightarrow \Omega ^2(\Sigma , {\text {At}} (P)^*)\times \mathcal {A}(\partial P),\ \ \theta \mapsto \big (-F^\theta \cdot s^\theta ,\ \partial \theta \big ). \end{aligned}$$

Here $F^\theta \in \Omega ^2(\Sigma ,\mathfrak {g}(P))$ is the curvature, and

38 $$\begin{aligned} s^\theta :{\text {At}}(P)=TP/G\rightarrow \mathfrak {g}(P) \end{aligned}$$

is the splitting (‘vertical projection’) defined by connection $\theta \in \mathcal {A}(P)$.

One obtains an affine moment map for the action of $^0\! {\text {Aut}} _+(P,\sigma )\subseteq  ^0\! {\text {Aut}} _+(P)$ by projection, replacing $s^\theta$ with $s^\theta |_{ {\text {At}} (P,\sigma )}$. We would like to interpret (35) as a symplectic reduction for this moment map. As we will see, the boundary term causes some complications. Let us therefore begin with the case $\partial {\Sigma }=\emptyset$.

### Symplectic structure: the case $\partial {\Sigma }=\emptyset$

If the boundary is empty, only the bulk term $-F^\theta \cdot s^\theta$ of the moment map remains. The moment map for ${\text {Aut}} _+(P,\sigma )$ is thus given by

39 $$\begin{aligned} \mathcal {A}^{{\text {pos}}}(P)\rightarrow \mathfrak {aut} (P,\sigma )^*=\Omega ^2(\Sigma , {\text {At}} (P,\sigma )^*),\ \theta \mapsto -F^\theta \cdot s^\theta |_{ {\text {At}} (P,\sigma )}. \end{aligned}$$

#### Proposition 6.2

Let $\Sigma$ be a compact oriented surface without boundary, and $P\rightarrow \Sigma$ a principal *G*-bundle with developing section satisfying $\textsf{e}(P,\sigma )=\chi (\Sigma )$. Then

$$\begin{aligned} {\text {Teich}}(\Sigma )=\mathcal {A}^{{\text {pos}}}(P)/\hspace{-2.97498pt}/ {\text {Aut}} _\textsf{o}(P,\sigma ), \end{aligned}$$

a symplectic quotient, with a residual action of ${\text {MCG}}(\Sigma )$ preserving the symplectic structure.

#### Proof

We claim that the zero level set of (39) is the space $\mathcal {A}^{{\text {pos}}}_{{\text {flat}}}(P)$ of flat connections. The map ${s^\theta } : {\text {At}} (P,\sigma )\rightarrow \mathfrak {g}(P)$ restricts to the identity on the subbundles $\mathfrak {g}(P,\sigma )$, and gives a commutative diagramwhere the vertical maps are the quotients maps for the subbundle $\mathfrak {g}(P,\sigma )$. For $\theta \in \mathcal {A}^{{\text {pos}}}(P)$ the lower horizontal map is an isomorphism, hence so is the upper map. Hence $F^\theta \cdot s^\theta (v)=0$ for all $v\in \mathfrak {aut} (P,\sigma )=\Gamma ( {\text {At}} (P,\sigma ))$ if and only if $F^\theta =0$. Since ${\text {Aut}} _+(P,\sigma )$ preserves the Atiyah–Bott form, the induced action of ${\text {Aut}} _+(P,\sigma )/ {\text {Aut}} _\textsf{o}(P,\sigma )\cong {\text {MCG}}(\Sigma )$ is again symplectic. $\square$

Since the condition $\textsf{e}(P)=\chi (\Sigma )$ determines *P* up to isomorphism, the symplectic 2-form $\omega$ on ${\text {Teich}}(\Sigma )$ does not depend on its choice. We will verify in Sect. 8 that this symplectic form is the standard Weil-Petersson form, given in Fenchel–Nielsen coordinates by Wolpert’s theorem.

It is clear from the construction that the residual action of the mapping class group ${\text {MCG}}(\Sigma )$ preserves the symplectic structure. Hence, we also have

#### Remark 6.3

By a classical result, obtained independently by Goldman [13] and Hitchin [16], the symplectic structure on Teichmüller space ${\text {Teich}}(\Sigma )$ may be obtained directly as a moduli space of flat connections, without having to invoke developing sections. That is,40for any choice of *G*-bundle with $\textsf{e}(P)=\chi (\Sigma )$. The proof of (40) is more involved; we do not know of an independent argument obtaining this result from Proposition 6.2.

### Symplectic structure: the case $\partial {\Sigma }\ne \emptyset$

We now turn to the case of a possibly non-empty boundary. We shall denote $Q=\partial P,\ \tau =\partial \sigma$. Equation (35) represents the Teichmüller space as a quotient by the group $^0\! {\text {Aut}} _\textsf{o}(P,\sigma )$, the identity component of automorphisms whose base map fixes $\partial {\Sigma }$. However:

The pullback of $\omega _{AB}$ to $\mathcal {A}^{{\text {pos}}}_{{\text {flat}}}(P)$ does not descend to the quotient.

The problem is that the restriction map $^0\! {\text {Aut}} _\textsf{o}(P,\sigma )\rightarrow {\text {Gau}} (Q)$ is non-trivial (in fact, it is surjective). Hence, the boundary terms of the moment map are present. Our strategy is to carry out the reduction in stages. Let

$$\begin{aligned} {\text {Aut}} _\textsf{o}(P,Q,\sigma )\subseteq  ^0\! {\text {Aut}} _\textsf{o}(P,\sigma ) \end{aligned}$$

be the kernel of the restriction map, and put

41 $$\begin{aligned} \widehat{{\text {Teich}}}(\Sigma )= \mathcal {A}^{{\text {pos}}}_{{\text {flat}}}(P)/ {\text {Aut}} _\textsf{o}(P,Q,\sigma )= {\text {Teich}}(\Sigma )\times _{{\text {Proj}}(\partial {\Sigma })} \mathcal {A}^{{\text {pos}}}(Q). \end{aligned}$$

Elements of this space are represented by hyperbolic structures on $\Sigma$ together with a lift of the corresponding projective structure on the boundary to a $\tau$-positive connection on *Q*.

#### Lemma 6.4

$\widehat{{\text {Teich}}}(\Sigma )$ is a symplectic quotient $\mathcal {A}^{{\text {pos}}}(P)/\hspace{-2.97498pt}/ {\text {Aut}} _\textsf{o}(P,Q,\sigma )$.

#### Proof

The Lie algebra $\mathfrak {aut} (P,Q,\sigma )$ is the space of sections of ${\text {At}} (P,\sigma )$ vanishing along the boundary $\partial {\Sigma }$. By the same argument as in the proof of Proposition 6.2, if $\theta \in \mathcal {A}^{{\text {pos}}}(P)$, we have $F^\theta \cdot s^\theta (v)=0$ for all $v\in \mathfrak {aut} (P,Q,\sigma )$ if and only if $F^\theta =0$. Hence, the symplectic quotient by this subgroup is $\widehat{{\text {Teich}}}(\Sigma )$.

According to Proposition 5.4, the map $^0\! {\text {Aut}} _\textsf{o}(P,\sigma )\longrightarrow {\text {Gau}} (Q,\tau )$ is surjective; hence $\widehat{{\text {Teich}}}(\Sigma )$ has a residual action of the group ${\text {Gau}} (Q,\tau )$, with quotient ${{\text {Teich}}}(\Sigma )$. Let

$$\begin{aligned} \mathcal {A}(Q,\tau )=\mathcal {A}(Q)/{\text {ann}}( \mathfrak {gau} (Q,\tau )). \end{aligned}$$

This is an affine ${\text {Gau}} (Q,\tau )$-space, with linear action the coadjoint action on $\mathfrak {gau} (Q,\tau )^*$.

#### Lemma 6.5

The moment map for the action of ${\text {Gau}} (Q,\tau )$ on $\widehat{{\text {Teich}}}(\Sigma )$ is given by

$$\begin{aligned} \Psi :\widehat{{\text {Teich}}}(\Sigma )\rightarrow \mathcal {A}(Q,\tau ),\ \ [\theta ]\mapsto \ \partial \theta \!\! \mod {\text {ann}}( \mathfrak {gau} (Q,\tau )). \end{aligned}$$

#### Proof

This follows by reduction, since the boundary term of the moment map for the $^0 {\text {Aut}} _\textsf{o}(P)$-action on $\mathcal {A}(P)$ is $\theta \mapsto \partial \theta$.

Note that $\Psi$ takes values in the subspace $\mathcal {A}^{{\text {pos}}}(Q,\tau )=\mathcal {A}^{{\text {pos}}}(Q)/{\text {ann}}( \mathfrak {gau} (Q,\tau ))$. It turns out that the image of this map is a single coadjoint orbit:

#### Lemma 6.6

The action of ${\text {Gau}} (Q,\tau )$ on $\mathcal {A}^{{\text {pos}}}(Q,\tau )$ is free and transitive.

#### Proof

This is a well-known fact from Drinfeld–Sokolov theory. We may assume that $Q=S^1\times G$, with $\tau (g,x)=g^{-1}\cdot 0$. The connections on *Q* are described by their connection 1-forms $A\in \Omega ^1(S^1,\mathfrak {g})$. Write

42 $$\begin{aligned} A=\left( \begin{array}{cc} {\frac{1}{2}}s &  a\\ u &  -{\frac{1}{2}}s \end{array} \right) \ {\textsf{d}}x \end{aligned}$$

with functions $a,s,u\in C^\infty (S^1)$; the connection is $\tau$-positive if and only if $a>0$. Taking the quotient by ${\text {ann}}( \mathfrak {gau} (Q,\tau ))\cong \Omega ^1(S^1,\mathfrak {n^-})$ amounts to omitting the lower left corner; hence *a*, *s* serve as parameters on $\mathcal {A}^{{\text {pos}}}(Q,\tau )$. There is a unique gauge transformation by an element $h\in C^\infty (S^1,B^-)= {\text {Gau}} (Q,\tau )$ putting (42) into Drinfeld–Sokolov normal form, that is, having 1 in the upper right corner and with vanishing diagonal entries. Explicitly,

$$\begin{aligned} h=\left( \begin{array}{cc} 1 &  0\\ -{\frac{1}{2}}s+{\frac{1}{2}}\frac{a'}{a}& 1 \end{array} \right) \left( \begin{array}{cc} a^{-\frac{1}{2}} &  0 \\ 0 &  a^{\frac{1}{2}} \end{array} \right) . \end{aligned}$$

In other words, *h* is the unique element taking the class of *A* in $\mathcal {A}^{{\text {pos}}}(Q,\tau )$ to the base point of $\mathcal {A}^{{\text {pos}}}(Q,\tau )$ given by $s=0,\ a=1$. $\square$

Since $\mathcal {O}=\mathcal {A}^{{\text {pos}}}(Q,\tau )$ is a coadjoint orbit, it has a unique symplectic structure such that the ${\text {Gau}} (Q,\tau )$-action is Hamiltonian, with moment map the inclusion. (See “Appendix A.1”.)

By the well-known ‘shifting trick’ from symplectic geometry, the quotient $\widehat{{\text {Teich}}}(\Sigma )/ {\text {Gau}} (Q,\tau )$ may be recast as a symplectic quotient. Let $\mathcal {O}^-$ be the space $\mathcal {O}$ with the opposite symplectic structure.

#### Theorem 6.7

The Teichmüller space is a symplectic quotient

$$\begin{aligned} {\text {Teich}}(\Sigma )=(\widehat{{\text {Teich}}}(\Sigma )\times \mathcal {O}^-)/\hspace{-2.97498pt}/ {\text {Gau}} (Q,\tau ), \end{aligned}$$

where $\widehat{{\text {Teich}}}(\Sigma )=\mathcal {A}^{{\text {pos}}}(P)/\hspace{-2.97498pt}/ {\text {Aut}} _\textsf{o}(P,Q,\sigma )$. In particular, ${\text {Teich}}(\Sigma )$ acquires a symplectic structure. The action of ${\text {MCG}}(\Sigma )$ on ${\text {Teich}}(\Sigma )$ preserves the symplectic structure.

#### Proof

Only the final claim remains to be proved. The action of $^b {\text {Aut}} _+(P,\sigma )$ on the space $\mathcal {A}^{{\text {pos}}}(P)$ preserves the Atiyah–Bott symplectic structure, and restricts to an action on the space of flat connections. We hence obtain a symplectic action of $^b {\text {Aut}} _+(P,\sigma )/ {\text {Aut}} _\textsf{o}(P,Q,\sigma )$ on $\widehat{{\text {Teich}}}(\Sigma )$. This contains ${\text {MCG}}(\Sigma )= {\text {Aut}} _+(P,Q,\sigma )/ {\text {Aut}} _\textsf{o}(P,Q,\sigma )$ and $^0 {\text {Aut}} _\textsf{o}(P,\sigma )/ {\text {Aut}} _\textsf{o}(P,Q,\sigma )= {\text {Gau}} (Q,\tau )$ as commuting subgroups. The moment map for the ${\text {Gau}} (Q,\tau )$-action is ${\text {MCG}}(\Sigma )$-invariant; hence we obtain a symplectic action of ${\text {MCG}}(\Sigma )$ on the quotient. $\square$

The symplectic structure obtained in this way does not depend on the choice of $(P,\sigma )$ subject to the condition $\textsf{e}(P,\sigma )=\chi (\Sigma )$, since any two choices are related by a bundle isomorphism (Proposition 4.8). The intermediate space $\widehat{{\text {Teich}}}(\Sigma )$ depends on the choice, but only through the boundary restriction $(Q,\tau )$. There is a canonical choice for the boundary restriction, and hence of the space $\widehat{{\text {Teich}}}(\Sigma )$, coming from the theory of Drinfeld–Sokolov reduction. We will discuss it in the next section, since it will also lead to a simpler description of ${\text {Teich}}(\Sigma )$.

#### Remark 6.8

It would be interesting to have a construction of the symplectic structure directly from the metric, as in the work of Tromba [35] (see also Donaldson [10], Diez-Ratiu [9]).

## ${\text {Teich}}(\Sigma )$ as a Hamiltonian Virasoro Space

We will now verify that the map ${\text {Teich}}(\Sigma )\rightarrow {\text {Proj}}(\partial {\Sigma })$, taking the equivalence class of a hyperbolic structure on a surface with boundary to the induced projective structure on the boundary, is an affine moment map. The affine structure on ${\text {Proj}}(\partial {\Sigma })$ comes from its identification with an affine subspace of the dual of the Virasoro Lie algebra $\mathfrak {vir}(\partial {\Sigma })$, at a suitable non-zero level. We will give explicit formulas for the Hill operator on the boundary, in terms of data coming from the hyperbolic 0-metric.

### Review of Hill operators and Virasoro algebra

We shall need some background material. For more detailed information, see the standard references [17, 25] as well as our earlier paper [3]. Let ${\textsf{C}}$ be a compact, oriented 1-manifold. For $r\in \mathbb {R}$, we denote by $|\Omega |_\textsf{C}^r$ the space of *r*-densities. A *k*-th order differential operator $D:|\Omega |_\textsf{C}^{r_1}\rightarrow |\Omega |_\textsf{C}^{r_2}$ has a principal symbol $\sigma _k(D)\in |\Omega |_\textsf{C}^{r_2-r_1-k}$. The principal symbol is scalar exactly when $r_2=r_1+k$. If $r_1+r_2=1$, the (formal) adjoint operator acts between the same spaces, and it makes sense to ask that *D* be self-adjoint. A *Hill operator* is a second order differential operator

$$\begin{aligned} L:|\Omega |_\textsf{C}^{-\frac{1}{2}}\rightarrow |\Omega |_\textsf{C}^{\frac{3}{2}} \end{aligned}$$

satisfying $L^*=L$ and $\sigma _2(L)=1$. The space of all Hill operators is an affine space ${\text {Hill}}(\textsf{C})$, with the space of quadratic differentials $|\Omega |^2_\textsf{C}$ as its space of translations. There is a ${\text {Diff}}_\textsf{o}(\textsf{C})$-equivariant isomorphism

43 $$\begin{aligned} {\text {Hill}}(\textsf{C}){\mathop {\longrightarrow }\limits ^{\cong }}{\text {Proj}}(\textsf{C}), \end{aligned}$$

taking a Hill operator *L* to the projective structure with charts $(u_1:u_2):U\rightarrow \mathbb {R}\!{\text {P}}(1)$, for local solutions $u_1,u_2\in |\Omega |_U^{-1/2}$ of $Lu=0$, with Wronskian $W(u_1,u_2)=-1$. The natural action of ${\text {Diff}}_+(\textsf{C})$ on ${\text {Hill}}(\textsf{C})$ is an affine action, with underlying linear action the coadjoint action. Here $|\Omega |^2_\textsf{C}$ is seen as the (smooth) dual to the space of vector fields ${\text {Vect}}(\textsf{C})=|\Omega |_\textsf{C}^{-1}$. The Virasoro algebra $\mathfrak {vir}(\textsf{C})$ is the central extension of ${\text {Vect}}(\textsf{C})$ defined by this action (see “Appendix A.1”). The action of ${\text {Diff}}_\textsf{o}(\textsf{C})$ on $\mathfrak {vir}^*_1(\textsf{C})$ is the coadjoint Virasoro action; see e.g. [8, 18, 19, 29, 36].

Using a local coordinate *x* on $\textsf{C}$, the *r*-density bundles are trivialized by the sections $|dx|^r$. In terms of this trivialization, a Hill operator takes on the form

44 $$\begin{aligned} L=\frac{d^2}{d x^2}+T(x) \end{aligned}$$

for a *Hill potential* *T*. For ${\textsf{F}}\in {\text {Diff}}(\textsf{C})$, the Hill operator ${\textsf{F}}^{-1}\cdot L$ has Hill potential ${\textsf{F}}^{-1}\cdot T$ given by the formula

45 $$\begin{aligned} ({\textsf{F}}^{-1}\cdot T)(x)={\textsf{F}}'(x)^2 T({\textsf{F}}(x))+{\frac{1}{2}}\mathcal {S}({\textsf{F}})(x) \end{aligned}$$

with the Schwarzian derivative [25, 26] $\mathcal {S}({\textsf{F}})=\frac{{\textsf{F}}'''}{{\textsf{F}}'}-\frac{3}{2}\left( \frac{{\textsf{F}}''}{{\textsf{F}}'}\right) ^2$. The map (43) factors through the *Drinfeld–Sokolov embedding*

46 $$\begin{aligned} {\text {Hill}}(\textsf{C})\rightarrow \mathcal {A}^{{\text {pos}}}(Q) \end{aligned}$$

for a canonically defined pair $(Q,\tau )$. The following coordinate-free description is due to Segal [29]. Let $|\Lambda |^{-1/2}_\textsf{C}$ be the bundle of $-{\frac{1}{2}}$ densities. A Hill operator *L* determines a linear connection on the 1-jet bundle

47 $$\begin{aligned} E=J^1(|\Lambda |^{-1/2}_\textsf{C}) \end{aligned}$$

with the property that $Lu=0$ if and only if $\nabla j^1(u)=0$. (By standard ODE theory, every solution is uniquely determined by its 1-jet at any given point.) Dually, we obtain a connection on $E^*$. Dualizing the projection $E\rightarrow |\Lambda |^{-1/2}_\textsf{C}$, we obtain a rank 1 subbundle of $E^*$, or equivalently a section of its projectivization. We take $Q\rightarrow \textsf{C}$ be the associated principal *G*-bundle, thus $\textsf{P}(E^*)=Q\times _G \mathbb {R}\!{\text {P}}(1)$, and let $\tau :Q\rightarrow \mathbb {R}\!{\text {P}}(1)\cong \partial \mathbb {D}$ be the map defining this section. The connection on $E^*$ defined by a Hill operator descends to a $\tau$-positive connection on *Q*, defining the inclusion (46). The image of the Drinfeld–Sokolov embedding will be called the Drinfeld–Sokolov slice, denoted

48 $$\begin{aligned} \mathcal {Z}\subseteq \mathcal {A}^{{\text {pos}}}(Q). \end{aligned}$$

The bundle $V_\tau =\tau ^*T\partial \mathbb {D}/G$ for Segal’s $(Q,\tau )$ is canonically isomorphic to the tangent bundle

$$\begin{aligned} V_\tau \cong T\textsf{C}. \end{aligned}$$

Hence, given a connection $\vartheta \in \mathcal {A}(Q)$, the map $\textsf{a}:T\textsf{C}\rightarrow V_\tau$ from (22) is scalar multiplication by a function *a*, and $\vartheta$ is positive if and only if $a>0$ everywhere. We have $\mathcal {Z}\subseteq a^{-1}(1)$.

The choice of a local coordinate *x* on $\textsf{C}$ determines a trivialization of $|\Lambda |_C^{-1/2}$, hence also of its jet bundle and consequently of *Q*. In this trivialization, $\tau (m,g)=g^{-1}\cdot 0$, and the Drinfeld–Sokolov embedding is given by the formula^2^

49 $$\begin{aligned} T\,|{\textsf{d}}x|^2\mapsto \left( \begin{array}{cc} 0 &  1\\ -T &  0 \end{array} \right) \ {\textsf{d}}x.\end{aligned}$$

From the coordinate-free description, it is clear that ${\text {Diff}}_+(\textsf{C})$ acts on *Q* by automorphisms preserving $\tau$, hence defining a splitting

50 $$\begin{aligned} {\text {Diff}}_+(\textsf{C})\rightarrow {\text {Aut}} _+(Q,\tau ). \end{aligned}$$

The Drinfeld–Sokolov embedding is equivariant for this action. Using local coordinates to trivialize the bundles, this is given by ${\textsf{F}}^{-1}\mapsto (h,{\textsf{F}}^{-1})\in C^\infty (\textsf{C},B^-)\rtimes {\text {Diff}}_+(\textsf{C})$ with

51 $$\begin{aligned} h= \left[ \begin{array}{cc} 1 & 0\\ \frac{1}{2}{\textsf{F}}'' ({\textsf{F}}')^{-1} &  1 \end{array}\right] \ \left[ \begin{array}{cc} ({\textsf{F}}')^{-\frac{1}{2}} & 0\\ 0 &  ({\textsf{F}}')^{\frac{1}{2}} \end{array}\right] . \end{aligned}$$

On may check directly that *h* is the unique $B^-$-valued function such that $h\bullet {\textsf{F}}^*A$ is again in the Drinfeld–Sokolov slice, with *T* replaced by ${\textsf{F}}^{-1}\cdot T$.

### Hill potential in terms of adapted coframes

Given a hyperbolic 0-metric $\textsf{g}$ on an oriented surface $\Sigma$ with boundary, we are interested in a description of the corresponding Hill operator in terms of adapted coordinates *x*, *y*. Let $\alpha _1,\alpha _2$ be an adapted orthonormal coframe for $\textsf{g}$, with associated spin connection $\kappa$. Write

$$\begin{aligned} \alpha _1=\frac{1}{y}(a(x){\textsf{d}}x+\ldots ),\ \ \alpha _2=\frac{{\textsf{d}}y}{y}+s(x){\textsf{d}}x+\ldots ,\ \ {\frac{1}{2}}(\alpha _1+\kappa )=y (u(x){\textsf{d}}x+\ldots ) \end{aligned}$$

where the dots indicate regular 1-forms whose pullback to the boundary vanishes.

#### Proposition 7.1

The Hill potential corresponding to the hyperbolic 0-metric $\textsf{g}$ is given by the formula

52 $$\begin{aligned} T={\frac{1}{2}}\left( \frac{a''}{a}-\frac{3}{2}\Big (\frac{a'}{a}\Big )^2\right) -au-\frac{1}{4}s^2-{\frac{1}{2}}\frac{a'}{a}s+{\frac{1}{2}}s'. \end{aligned}$$

#### Proof

The 0-connection 1-form (14) reads

$$\begin{aligned} \left( \begin{array}{cc} \frac{1}{2y}\ {\textsf{d}}y +{\frac{1}{2}}s(x){\textsf{d}}x+\ldots &  \frac{1}{y} (a(x){\textsf{d}}x+\ldots )\\ y(u(x){\textsf{d}}x+\ldots )&  -\frac{1}{2y}\ {\textsf{d}}y -{\frac{1}{2}}s(x){\textsf{d}}x+\ldots \end{array}\right) . \end{aligned}$$

In Sect. 4.3, we explained that the *regular* connection 1-form, describing $\partial \theta \in \mathcal {A}^{{\text {pos}}}(Q)$, is obtained by applying the ‘singular gauge transformation’ by ${\text {diag}}(y^{{\frac{1}{2}}},y^{-{\frac{1}{2}}})$, and pulling back to $y=0$. The result is the connection 1-form (42) from the proof of Lemma 6.6. By working out the gauge transformation indicated there, taking the connection to Drinfeld–Sokolov normal form, one obtains *T* as minus the lower left corner. The result of this straightforward calculation is (52). $\square$

#### Example 7.2

The Hill potential for the Poincaré disk, with coordinates $\phi$ as in Example 3.1b, is $T(x)=\frac{1}{4}$. For the trumpet, with geodesic neck of length $\ell$ (Example 3.1c), we obtain $T(x)=-\frac{1}{4}\ell ^2$. For the Fefferman–Graham coframe (Example 3.1d), the Hill potential agrees with the function *T* given in that formula.

### Hill potential in terms of geodesic curvature

We will now give a second description of the Hill operator of a hyperbolic 0-metric $\textsf{g}$, motivated by the discussion in Maldacena-Stanford-Yang [20, Section 3]. Observe that the function *a*(*x*) in (52) may be read off from the leading term of the volume form;

$$\begin{aligned} {\textsf{d}} {\text {vol}}_{\textsf{g}}=\frac{1}{y^2} \big (a(x)+O(y^1)\big ) {\textsf{d}}x\wedge {\textsf{d}}y. \end{aligned}$$

For $y>0$, let *k*(*x*, *y*) be the geodesic curvature of the curve $t\mapsto (x+t,y)$. Recall that for the standard hyperbolic metric on the upper half plane, the horizontal lines all have geodesic curvature equal to 1. Hence $k(x,y)=1$ for all $x,y\in \mathbb {H}$. It turns out that in general, $k(x,y)=1+O(y^2)$:

#### Lemma 7.3

For every hyperbolic 0-metric, the limit

$$\begin{aligned} c(x)=\lim _{y\rightarrow 0} \frac{k(x,y)-1}{y^2} \end{aligned}$$

exists and defines a smooth function of *x*.

#### Proof

By Theorem 3.9, the hyperbolic 0-metric may be written $\textsf{g}=\frac{1}{g^2}({\textsf{d}}f^2+{\textsf{d}}g^2)$ for functions *f*, *g* with $\frac{\partial f}{\partial x}(x,0)>0$ and $g(x,0)=0,\ \frac{\partial g}{\partial y}(x,0)>0$.

That is, (*f*, *g*) defines a local isometry to $\overline{\mathbb {H}}$. The image of the curve $t\mapsto (x+t,y)$ under this isometry is the curve $t\mapsto (f(x+t,y),g(x+t,y))$ in $\mathbb {H}$; its geodesic curvature *k*(*x*, *y*) is computed as

$$\begin{aligned} k=\frac{f'}{\big ((f')^2+(g')^2\big )^{1/2}}+f \frac{f' g''-f'' g'}{\big ((f')^2+(g')^2\big )^{3/2}}, \end{aligned}$$

where the prime denotes *x*-derivatives. Substituting Taylor series

$$\begin{aligned} f(x,y)\sim \sum _i f_i(x)y^i,\ g(x,y)\sim \sum _i g_i(x) y^i\end{aligned}$$

one finds, by direct but somewhat lengthy calculation,

$$\begin{aligned} k(x,y)=1+c(x) y^2+y^3 \end{aligned}$$

with

53 $$\begin{aligned} c=\frac{g_1 g_1''}{(f_0')^2}-\frac{g_1 g_1' f_0''}{(f_0')^3}-{\frac{1}{2}}\frac{(g_1')^2}{(f_0')^2}. \end{aligned}$$

(The calculation requires writing *f*, *g* up to second order, but $f_1,f_2,g_2$ do not enter the final expression.) $\square$

#### Theorem 7.4

The Hill potential is given by

54 $$\begin{aligned} T={\frac{1}{2}}\left( \frac{a''}{a}-\frac{3}{2}\big (\frac{a'}{a}\big )^2\right) +\frac{a^2}{2} c \end{aligned}$$

where *c* is obtained from the limit of the geodesic curvatures of the curves $t\mapsto (x+t,y)$ as $c(x)=\lim _{y\rightarrow 0}(k(x,y)-1)/y^2$.

#### Proof

Continuing the notation from the proof of Lemma 7.3, we may take $\alpha _1=\frac{{\textsf{d}}f}{g},\ \alpha _2=\frac{{\textsf{d}}g}{g}$ as an adapted orthonormal coframe. Using the Taylor expansion of *f*, *g*, we find

$$\begin{aligned} \alpha _1=\frac{1}{y} (\frac{f_0'}{g_1}{\textsf{d}}x+\ldots ),\ \ \ \alpha _2=\frac{{\textsf{d}}y}{y}+\frac{g_1'}{g_1}{\textsf{d}}x+\ldots ,\ \ \alpha _1+\kappa =0 \end{aligned}$$

where dots indicate terms that pull back to zero on the boundary $y=0$. Hence, the functions *a*, *s*, *u* are given by

$$\begin{aligned} a=\frac{f_0'}{g_1},\ \ s=\frac{g_1'}{g_1},\ \ u=0. \end{aligned}$$

Using the formula (53) for *k*(*x*), this gives

$$\begin{aligned} c=\frac{1}{a^2} \Big (s'-\frac{a'}{a}s-\frac{1}{2}s^2\Big ). \end{aligned}$$

Now use (52). $$\square $$

### Verifying the moment map condition

We are now in position to describe the moment map for the $\widetilde{{\text {Diff}}}_\textsf{o}(\partial {\Sigma })$-action on the infinite-dimensional Teichmüller space.

#### Theorem 7.5

The action of $\widetilde{{\text {Diff}}}_\textsf{o}(\partial {\Sigma })$ on ${\text {Teich}}(\Sigma )$ is Hamiltonian, with moment map

$$\begin{aligned} \Phi :{\text {Teich}}(\Sigma )\rightarrow \mathfrak {vir}^*_{-1}(\partial \Sigma ),\ \ [\textsf{g}]\mapsto -L \end{aligned}$$

taking the equivalence class of a hyperbolic structure to minus the Hill operator for the associated projective structure on the boundary.

#### Remark 7.6

We obtain a moment map at level $-1$ due to our specific choice of metric $\xi \cdot \eta ={\text {tr}}(\xi \eta )$ on $\mathfrak {g}$. Multiplying the metric by a nonzero factor, the symplectic form and moment map (and in particular its level) scale accordingly.

Our starting point is the description (Theorem 6.7)where $(Q,\tau )$ is the boundary restriction of $(P,\sigma )$. We shall take this boundary restriction to be Segal’s bundle from Sect. 7.1. Denote $\textsf{C}=\partial {\Sigma }$.

The action of $\widetilde{{\text {Diff}}}_\textsf{o}(\textsf{C})$ is obtained as a quotient of the action of (a cover of) ${\text {Aut}} _\textsf{o}(Q,\tau )$ on both spaces, $\widehat{{\text {Teich}}}(\Sigma )$ and $\mathcal {O}$. Recall that for Segal’s bundle, there is a canonical splitting ${\text {Diff}}_\textsf{o}(\textsf{C})\rightarrow {\text {Aut}} _\textsf{o}(Q,\tau )$. This lifts to the universal covering. Hence, we may compute the ${\text {Diff}}_\textsf{o}(\textsf{C})$-part of the moment map on both spaces.

The choice of a a coordinate *x* on the boundary gives a trivialization $Q=\textsf{C}\times G$. In terms of this trivialization, the connection $\partial \theta$ is described by a connection 1-form *A* as in (42).

#### Proposition 7.7

The moment map for the $\widetilde{{\text {Diff}}}_\textsf{o}(\partial {\Sigma })$-action on $\widehat{{\text {Teich}}}(\Sigma )$ is given in coordinates by

55 $$\begin{aligned} \widehat{{\text {Teich}}}(\Sigma )\rightarrow |\Omega |^2_{\partial {\Sigma }},\ \ [\theta ]\mapsto \big (-\frac{1}{2} s' + \frac{1}{4} s^2+au-\frac{1}{2} a'')\ |{\textsf{d}}x|^2.\end{aligned}$$

Here the functions *a*, *u*, *s* are defined by (42).

#### Proof

The boundary term of the moment map is given by $[\theta ]\mapsto (A,{\frac{1}{2}}{\text {tr}}(A^2))\in \Omega ^1(\textsf{C},\mathfrak {g})\times |\Omega |^2_\textsf{C}$; see “Appendix B”. On the other hand, the coordinate expression of the inclusion ${\text {Vect}}(\textsf{C})\rightarrow \mathfrak {gau} (Q,\tau )\rtimes {\text {Vect}}{\textsf{C}}$ is given by the infinitesimal version of (51)^3^

$$\begin{aligned} f\,\frac{\partial }{\partial x}\mapsto \left( \left( \begin{array}{cc} {\frac{1}{2}}f' & 0\\ -\frac{1}{2}f'' &  -\frac{1}{2} f' \end{array}\right) ,\ f\,\frac{\partial }{\partial x}\right) . \end{aligned}$$

Using the expression (42) for *A*, the corresponding component of the moment map is

$$\begin{aligned} \int _{S^1} {\text {tr}}\left( \left( \begin{array}{cc} {\frac{1}{2}}s &  a\\ u &  -{\frac{1}{2}}s \end{array} \right) \,\left( \begin{array}{cc}{\frac{1}{2}}f' &  0\\ -{\frac{1}{2}}f'' &  -{\frac{1}{2}}f' \end{array}\right) \right) \ +{\frac{1}{2}}\int _{S^1} {\text {tr}}\left( \begin{array}{cc} {\frac{1}{2}}s &  a\\ u &  -{\frac{1}{2}}s \end{array} \right) ^2 f \\ =\int _{S^1}\left( {\frac{1}{2}}sf'-{\frac{1}{2}}af''+\frac{1}{4}sf+auf \right) =\int _{S^1} \left( - {\frac{1}{2}}s'-{\frac{1}{2}}a''+\frac{1}{4} s^2+au \right) f. \end{aligned}$$

$$\square $$

We recognize some, but not all, of the terms in the formula (52) for the Hill potential. One expects to obtain the remaining terms from a calculation of the $\widetilde{{\text {Diff}}}_\textsf{o}(\textsf{C})$-moment map on $\mathcal {O}$. Through explicit calculation, we checked that this is indeed the case, thereby obtaining a proof of Theorem 7.5. However, there is a much simpler argument, using the Drinfeld–Sokolov slice:

#### Proof of Theorem 7.5

Recall that the moment map $\Psi :\widehat{{\text {Teich}}}(\Sigma )\rightarrow \mathcal {A}(Q,\tau )$ is given by $[\theta ]\mapsto \partial \theta \mod {\text {ann}} \mathfrak {gau} (Q,\tau )$, and the set of all $\partial \theta \mod {\text {ann}} \mathfrak {gau} (Q,\tau )$ is a single coadjoint orbit $\mathcal {O}=\mathcal {A}^{{\text {pos}}}(Q,\tau )$. The Drinfeld–Sokolov slice $\mathcal {Z}\subseteq \mathcal {A}^{{\text {pos}}}(Q)$ descends to a slice for the ${\text {Gau}} (Q,\tau )$-action on $\mathcal {O}=\mathcal {A}^{{\text {pos}}}(Q)/{\text {ann}}(Q,\tau )$, consisting of just a single point, $\mu _0\in \mathcal {O}$, and the stabilizer of this point under ${\text {Gau}} (Q,\tau )$ is *trivial*. (See Lemma 6.6.)

Letting $\mu _0$ be the corresponding point in $\mathcal {O}$, we have

$$\begin{aligned} {\text {Teich}}(\Sigma )=\Psi ^{-1}(\mu _0)\subseteq \widehat{{\text {Teich}}}(\Sigma ) \end{aligned}$$

as a symplectic submanifold. The moment map for the $\widetilde{{\text {Diff}}}_\textsf{o}(\textsf{C})$-action on ${\text {Teich}}(\Sigma )$ may be computed by restricting the moment map to this cross-section.

Using coordinates, as above, $\mathcal {Z}$ is given by $a=1,s=0,u=-T$ where *T* is the Hill potential. (The point $\mu _0\in \mathcal {O}$ is the point given by $a=1,s=0$). Hence, on $\Psi ^{-1}(\mu _0)$ the moment map restricts to $[\theta ]\mapsto -T$. $\square$

## The Symplectic form in Fenchel–Nielsen Coordinates

### Fenchel–Nielsen parameters

Let $\Sigma$ be a compact, connected, oriented surface (possibly with boundary), of negative Euler characteristic $\chi (\Sigma )<0$. The construction of Fenchel–Nielsen parameters on ${\text {Teich}}(\Sigma )$ for surfaces without boundary is well-explained in [11]; we describe a straightforward generalization to the case of a possibly non-empty boundary.

Recall first that every simple, closed curve $\textsf{D}\subseteq \Sigma$, neither contractible nor homotopic to a boundary component, determines a *twist flow* $\mathbb {R}\times {\text {Teich}}(\Sigma )\rightarrow {\text {Teich}}(\Sigma )$: Given $\textsf{g}$, one obtains a new metric $\textsf{g}_\tau$ by cutting the surface along the geodesic homotopic to $\textsf{D}$, and gluing the two sides back together after rotating (twisting) one of the ends by an amount $\tau$.

#### Remark 8.1

A more detailed description: Given $[\textsf{g}]\in {\text {Teich}}(\Sigma )$, choose a representative $\textsf{g}$ having $\textsf{D}$ as a closed geodesic. A collar neighborhood *U* of $\textsf{D}$ is isometric to a neighborhood of the geodesic of a hyperbolic cylinder (see Sect. 2.4.2), and so is isometric to $\textsf{D}\times (-\epsilon ,\epsilon )$ with the hyperbolic metric (8). For any $\tau \in \mathbb {R}$, we obtain a new hyperbolic metric $\textsf{g}_\tau$ by letting $\textsf{g}_\tau |_{\Sigma -U}=\textsf{g}|_{\Sigma -U}$ and taking $\textsf{g}_\tau |_U$ to be the pullback of $\textsf{g}|_U$ under the diffeomorphism

56 $$\begin{aligned} (x,u)\mapsto \big (x+\frac{\tau }{\ell } f(u),u\big ), \end{aligned}$$

where $f(u)=0$ for $u<-\frac{1}{2}\epsilon$ and $f(u)=1$ for $u>{\frac{1}{2}}\epsilon$. The twist flow is given by $[\textsf{g}]\mapsto [\textsf{g}_\tau ]$.

Since $\chi (\Sigma )<0$, we may choose a pairs-of-pants decomposition of $\Sigma$. There are $2g-2+r=-\chi (\Sigma )$ distinct pants; their boundary curves consist of the boundary loops $\textsf{C}_j,\ j=1,\ldots ,r$ of $\Sigma$ and $3g-3+r$ simple closed curves $\textsf{D}_i\subseteq {\text {int}}(\Sigma )$.

Each of the $\textsf{D}_i$ defines a length parameter $\ell _i>0$ (the length of the unique closed geodesic homotopic to $\textsf{D}_i$), as well as a twist flow. In addition, each boundary component $\textsf{C}_j$ determines a length parameter $\ell _j$ (given by the length of the unique geodesic of $[\textsf{g}]$ homotopic to the ideal boundary $\textsf{C}_j$) as well as an action of $\widetilde{{\text {Diff}}}_\textsf{o}(\textsf{C}_j)$ (coming from the action of diffeomorphisms in $^b {\text {Diff}}_\textsf{o}(\Sigma )$ that are supported in collar neighborhoods of the $\textsf{C}_j$). These actions on ${\text {Teich}}(\Sigma )$ all commute, with quotient $\mathbb {R}_{>0}^{3g-3}\times \mathbb {R}_{>0}^r$ given by the length parameters. This action has a global slice, determined by the choice of a system of *model seams*. Choose an embedded 1-dimensional submanifold $E\subseteq \Sigma$ with boundary $\partial E\subseteq \partial \Sigma$, in such a way for any two distinct boundary circles of a given pants *P*, there is a unique component of $P\cap E$ connecting those two boundary components. We also assume that $P\cap E$ meets these boundary components transversely. The three components of $P\cap E$ are the model seams for the pair of pants *P*. Finally, choose orientation preserving parametrizations $\textsf{C}_j\cong S^1$, such that $\partial E\cap \textsf{C}_j$ maps to the antipodal points $\{-1,1\}\in S^1\subseteq \mathbb {C}$.

The desired slice consists of all $[\textsf{g}]\in {\text {Teich}}(\Sigma )$, where $\textsf{g}$ is a hyperbolic 0-metric such that (i) all $\textsf{D}_i$ are geodesics, (ii) the connected components of $E-\partial E\subseteq {\text {int}}(\Sigma )$ are geodesics, (iii) the projective structure on the boundary components $\textsf{C}_j$ is constant (i.e., $S^1$-equivariant). This gives an identification

57 $$\begin{aligned} {\text {Teich}}(\Sigma )\cong (\mathbb {R}_{>0}\times \mathbb {R})^{3g-3+r}\times \prod _{j=1}^r (\mathbb {R}_{>0}\times \widetilde{{\text {Diff}}}_\textsf{o}(S^1)) \end{aligned}$$

with the slice given as $(\mathbb {R}_{>0}\times 0)\times \prod _{j=1}^r (\mathbb {R}_{>0}\times {\text {Id}})^r$. Denote the corresponding parameters by $\ell _i,\tau _i,\ell _j,{\textsf{F}}_j$; we choose the parametrization in such a way that the *i*-th twist flow is given by $\tau _i\mapsto \tau _i+\tau$ (leaving all other parameters unchanged) and the *j*-th action of ${\textsf{F}}\in \widetilde{{\text {Diff}}}_\textsf{o}(S^1)$ is given by ${\textsf{F}}_j\mapsto {\textsf{F}}_j\circ {\textsf{F}}^{-1}$ (leaving all other parameters unchanged).

### Related Teichmüller spaces

Given a hyperbolic 0-metric $\textsf{g}$ on $\Sigma$, each boundary component $\textsf{C}_j$ determines a unique simple, closed geodesic $\textsf{C}_j'\subseteq \Sigma$ homotopic to $\textsf{C}_j$; this is the geodesic end of the *j*-th boundary trumpet. Removing the trumpets creates a surface $\Sigma '$ with geodesic boundary $\sqcup _{j}\textsf{C}_j'$, called the *compact core* of $\Sigma$. Of course, $\Sigma '$ is diffeomorphic to $\Sigma$ (as a surface with boundary). The map

58 $$\begin{aligned} {\text {Teich}}(\Sigma )\longrightarrow {\text {Teich}}_{{\text {geod}}}(\Sigma ),\ \ \ \end{aligned}$$

taking the equivalence class of a hyperbolic 0-metric on $\Sigma$ to the equivalence class of the (ordinary) hyperbolic metric on $\Sigma$ with *geodesic boundary*, is the quotient maps for the action of $\prod _{i=1}^r \widetilde{{\text {Diff}}}_\textsf{o}(\textsf{C}_i)$. The corresponding Fenchel–Nielsen description just omits the $\widetilde{{\text {Diff}}}_\textsf{o}(S^1)$-factors in (57). Fixing the lengths $b_j$ of the boundary components, one obtains the space ${\text {Teich}}_{{\text {geod}}}(\Sigma ,b_1,\ldots ,b_r)$. As another variation, having chosen parametrizations $\textsf{C}_j\cong S^1$ of the boundary components, we may consider the subspace

$$\begin{aligned} {\text {Teich}}_{{\text {bordered}}}(\Sigma )\subseteq {\text {Teich}}(\Sigma ) \end{aligned}$$

for which the projective structure on each $\textsf{C}_j$ is ‘constant’ (i.e., invariant under rigid rotations). On this subspace, we have a residual action of $\mathbb {R}^r$, where the *j*-th copy of $\mathbb {R}$ rotates the *j*-th boundary component. This version of the Teichmüller space may be interpreted as a space of hyperbolic metrics with geodesic boundary, together with a ‘marking’ on each boundary component. This space of ‘bordered’ hyperbolic metrics appears in Mirzakhani’s work, see [24, Section 4]. The Fenchel–Nielsen description becomes

$$\begin{aligned} {\text {Teich}}_{{\text {bordered}}}(\Sigma )\cong (\mathbb {R}_{>0}\times \mathbb {R})^{3g-3+2r}. \end{aligned}$$

One can also consider mixtures of such spaces, e.g., taking some boundary components to be ideal boundaries (with $\varrho ^{-2}$-boundary behaviour of the metric), other boundaries as marked geodesic boundaries.

### Teichmüller space of the trumpet

Let $\widetilde{N}$ denote the Teichmüller space of hyperbolic 0-metrics on $S^1\times [-\infty ,\infty ]$, such that the induced projective structure on the left boundary $S^1\times \{-\infty \}$ is constant, and denote by *N* the corresponding Riemann moduli space. As explained above, *N* may also be regarded as a moduli space of hyperbolic metrics on $S^1\times [0,\infty )$ for which $S^1\times \{0\}$ is a geodesic, of some length $\ell >0$, while $\textsf{g}$ has the boundary behaviour of a 0-metric along the ideal boundary $S^1\times \{\infty \}$. As a space,

59 $$\begin{aligned} N=\mathbb {R}_{>0}\times {\text {Diff}}_\textsf{o}(S^1) \end{aligned}$$

where the $\mathbb {R}_{>0}$ factor indicates the length $\ell$ of the geodesic boundary. This space comes with an action of ${\text {Diff}}_\textsf{o}(S^1)$ by ${\textsf{F}}_1\cdot (\ell ,{\textsf{F}})=(\ell ,{\textsf{F}}\circ {\textsf{F}}_1^{-1})$ and an action of $S^1=\mathbb {R}/\mathbb {Z}$ by $t\cdot (\ell ,{\textsf{F}})\mapsto (\ell ,{\textsf{F}}+t)$. (Here multiplication on $S^1=\mathbb {R}/\mathbb {Z}$ is written additively.) The following result describes the symplectic structure on *N*; the 2-form on $\widetilde{N}$ is obtained by pullback. (For a more conceptual explanation of the formula, see “Appendix C”.)

For $\ell >0$, we have the Hill operator $L(\ell )=\frac{d^2}{d x^2}+T(\ell )$ with the constant Hill potential $T(\ell )=-\frac{1}{4}\ell ^2\in {\text {Hill}}(S^1)$.

#### Theorem 8.2

(Trumpet). The space (59) has a unique invariant symplectic form $\omega _N$, in such a way that the ${\text {Diff}}_\textsf{o}(S^1)$ is Hamiltonian, with moment map $(\ell ,{\textsf{F}})\mapsto -{\textsf{F}}^{-1}\cdot L(\ell )\in {\text {Hill}}(S^1)$. This 2-form is given by the formula

60 $$\begin{aligned} \omega _N=-\frac{1}{4}{\textsf{d}}\int _{S^1}\big (\ell ^2{\textsf{F}}'\,{\textsf{d}}{\textsf{F}}+({\textsf{F}}')^{-1}{\textsf{d}}{\textsf{F}}'' \big ) \end{aligned}$$

The $S^1$-action is Hamiltonian as well, with moment map $(\ell ,{\textsf{F}})\mapsto \frac{1}{4}\ell ^2$. The symplectic quotient at $\frac{1}{4}\ell ^2$ for the latter action is the coadjoint Virasoro orbit through $L(\ell )$ (with the opposite symplectic structure).

Before proving this result, we have to explain the ingredients of (60). For fixed $x\in S^1$, we have the evaluation map ${\text {ev}}_x:{\text {Diff}}(S^1)\rightarrow \mathbb {R}/\mathbb {Z},\ {\textsf{F}}\mapsto {\textsf{F}}(x)$. As in [3] we shall denote this function on ${\text {Diff}}(S^1)$ simply by ${\textsf{F}}(x)$ (thinking of ${\textsf{F}}$ as a variable). The exterior derivative ${\textsf{d}}({\textsf{F}}(x))$ of this function is a 1-form on ${\text {Diff}}(S^1)$; letting *x* vary this is a 1-form on diffeomorphisms with values in periodic functions,

$$\begin{aligned} {\textsf{d}}\textsf{F}\in \Omega ^1({\text {Diff}}_\textsf{o}(S^1),|\Omega |^0_{S^1}). \end{aligned}$$

On the other hand, for fixed $\textsf{F}$ we may take the exterior derivative of the function $x\mapsto \textsf{F}(x)$. We shall denote it by

$$\begin{aligned} {\textsf{F}}'\in \Omega ^0({\text {Diff}}_\textsf{o}(S^1),|\Omega |^1_{S^1}) \end{aligned}$$

(a more accurate notation would be ${\textsf{F}}'(x)|{\textsf{d}}x|$). Higher derivatives are defined as well; for example, ${\textsf{F}}''$ is naturally a function on ${\text {Diff}}_\textsf{o}(S^1)$ with values in quadratic differentials. Since ${\textsf{F}}'(x)>0$ everywhere, we may also consider $1/{\textsf{F}}'\in \Omega ^0({\text {Diff}}_\textsf{o}(S^1),|\Omega |^{-1}_{S^1})$. With this understanding, each of the terms in (60) is a 2-form on ${\text {Diff}}_\textsf{o}(S^1)$ with values in $|\Omega |^{1}_{S^1}$; integration of the 1-density over $S^1$ results in a 2-form on ${\text {Diff}}_\textsf{o}(S^1)$.

#### Proof of Theorem 8.2

Expanding (60), we have

61 $$\begin{aligned} \omega _N=\frac{1}{4} \int _{S^1}\ \ \Big ( -{\textsf{F}}' {\textsf{d}}\ell ^2\wedge {\textsf{d}}\textsf{F}-\ell ^2\,{\textsf{d}}{\textsf{F}}'\wedge {\textsf{d}}{\textsf{F}}+\frac{{\textsf{d}}{\textsf{F}}'\wedge {\textsf{d}}{\textsf{F}}''}{({\textsf{F}}')^2} \Big ). \end{aligned}$$

To check that it does satisfies the moment map condition, we consider its contraction with a left-invariant vector field $v^L$ on ${\text {Diff}}_\textsf{o}(S^1)$ corresponding to $v\in {\text {Vect}}(S^1)$. The flow of $v^L$ on ${\text {Diff}}_\textsf{o}(S^1)$ is given in terms of the flow $t\mapsto \exp (tv)$ by ${\textsf{F}}\mapsto {\textsf{F}}\circ \exp (-tv)$. As explained in [3, Lemma 4.8], if $v=f(x)\partial _x$ then

$$\begin{aligned} \iota (v^L){\textsf{d}}{\textsf{F}}=-{\textsf{F}}'\, f.\end{aligned}$$

The contractions with ${\textsf{d}}{\textsf{F}}',\ {\textsf{d}}{\textsf{F}}''$ are obtained by taking derivatives of this expression. Hence,

$$\begin{aligned} \iota (v^L)\omega _N=\frac{1}{4} \int _{S^1} \Big (-({\textsf{F}}')^2 f {\textsf{d}}\ell ^2-\ell ^2 f {\textsf{F}}'{\textsf{d}}{\textsf{F}}' -\ell ^2(-{\textsf{F}}'f)'{\textsf{d}}{\textsf{F}}+\frac{ (-{\textsf{F}}' f)' {\textsf{d}}{\textsf{F}}''-(-{\textsf{F}}' f)'' {\textsf{d}}{\textsf{F}}'}{({\textsf{F}}')^2} \Big ) \end{aligned}$$

Use integration by parts so that no derivatives of *f* appear:

$$\begin{aligned} \iota (v^L)\omega _N=\frac{1}{4} \int _{S^1} \Big (-({\textsf{F}}')^2 {\textsf{d}}\ell ^2-2\ell ^2 {\textsf{F}}'{\textsf{d}}{\textsf{F}}' +{\textsf{F}}'\Big ( \frac{{\textsf{d}}{\textsf{F}}''}{({\textsf{F}}')^2} \Big )'+{\textsf{F}}'\Big ( \frac{{\textsf{d}}{\textsf{F}}'}{({\textsf{F}}')^2}\Big )'' \Big )f. \end{aligned}$$

After simplifications, this becomes

$$\begin{aligned} \iota (v^L)\omega _N={\textsf{d}}\int _{S^1} \Big (\big (-\frac{1}{4} (F')^2 {\textsf{d}}\ell ^2 +\frac{1}{2} \mathcal {S}({\textsf{F}})\big ) \Big ) f ={\textsf{d}}\int _{S^1} \big ({\textsf{F}}^{-1}\cdot T(\ell )\big )\,f. \end{aligned}$$

where $T(\ell )=-\frac{1}{4}\ell ^2$ is the Hill potential corresponding to $\ell$. This shows that $(\ell ,{\textsf{F}})\mapsto -{\textsf{F}}^{-1}\cdot T(\ell )$ is a moment map for the action. Consider on the other hand the $S^1$-action ${\textsf{F}}\mapsto {\textsf{F}}+t\mod \mathbb {Z}$. Letting *Z* denote its generating vector field, we have $\iota _Z {\textsf{d}}{\textsf{F}}=1$, hence $\iota _Z{\textsf{d}}{\textsf{F}}'=0,\ \iota _Z{\textsf{d}}{\textsf{F}}''=0$. It follows that

$$\begin{aligned} \iota (Z)\omega _N=\frac{1}{4}\int _{S^1} {\textsf{F}}' {\textsf{d}}\ell ^2=\frac{1}{4} {\textsf{d}}\ell ^2 \end{aligned}$$

where we used $\int _{S^1}{\textsf{F}}'=1$ by fundamental theorem of calculus. It follows that $(\ell ,{\textsf{F}})\mapsto -\frac{1}{4}\ell ^2$ is a moment map for this action. The reduction of *N* with respect to this $S^1$-action, at level $-\frac{1}{4}\ell ^2$, is ${\text {Diff}}_+(S^1)/S^1$ with a closed ${\text {Diff}}_\textsf{o}(S^1)$-invariant 2-form whose moment map gives a bijection onto ${\text {Diff}}_\textsf{o}(S^1)\cdot L(\ell )\subseteq \mathfrak {vir}^*_1(S^1)$. The reduction hence equals the (hyperbolic) coadjoint Virasoro orbit through $L(\ell )$. The fact that all the $S^1$-reduced spaces of $(N,\omega _N)$ are symplectic implies that $\omega _N$ must itself be symplectic. The uniqueness part for $\omega _N$ follows since the difference of two 2-forms on *N* satisfying the moment map condition is basic for the ${\text {Diff}}_\textsf{o}(S^1)$-action, and hence is zero since the quotient is 1-dimensional.

### Fenchel–Nielsen description of the symplectic form

For $i=1,\ldots ,3g-3+r$, let $\ell _i,\tau _i$ be the length and twist parameters with respect to $\textsf{D}_i$, thought of as functions on ${\text {Teich}}(\Sigma )$. Also, for $j=1,\ldots ,r$ let

$$\begin{aligned} \pi _j:{\text {Teich}}(\Sigma )\rightarrow N\end{aligned}$$

be the map given by projection to the *j*-th boundary factor in (57) (the Teichmüller space of the *j*-th trumpet), followed by the quotient map $\widetilde{N}\rightarrow N$.

#### Theorem 8.3

In terms of Fenchel–Nielsen parameters (57), the symplectic form on ${\text {Teich}}(\Sigma )$ is given by

62 $$\begin{aligned} \omega = {\frac{1}{2}}\sum _{i=1}^{3g-3+r} {\textsf{d}}\ell _i\wedge {\textsf{d}}\tau _i+\sum _{j=1}^r \pi _j^*\omega _{N}. \end{aligned}$$

In the case without boundary, this is the well-known Wolpert formula [37] for the Weil-Petersson symplectic form. The first part of the following argument is adapted from [27, Section 3.3.2].

#### Proof

We verify the formula at any given $[\textsf{g}]\in {\text {Teich}}(\Sigma )$. Pick a representative $\textsf{g}$ as in Sect. 8.1; in particular, the $\textsf{D}_i$ are geodesics. It suffices to verify the formula on tangent vectors of the following types: infinitesimal changes of length or twist parameters for the curves $\textsf{D}_i$, tangent vectors $v\in {\text {Vect}}(\textsf{C}_j)$ corresponding to the $\widetilde{{\text {Diff}}}_\textsf{o}(\textsf{C}_j)$-factors, as well as infinitesimal changes of the length parameters for $\textsf{C}_j'$, the geodesic ends of the trumpets. These tangent vectors are realized by variations of the hyperbolic metric $\textsf{g}$.

Consider a fixed $\textsf{D}=\textsf{D}_i$. We may introduce coordinates on some collar neighborhood of $\textsf{D}$ so that $\textsf{g}$ is given by the metric of the hyperbolic cylinder, (8), with the coframe (Example 3.1c)

$$\begin{aligned} \alpha _1=\cosh (u)\ell \ {\textsf{d}}x,\ \ \alpha _2=-{\textsf{d}}u,\ \ \kappa =-\sinh (u)\ell \ {\textsf{d}}x \end{aligned}$$

and corresponding connection one-form

$$\begin{aligned} A={\frac{1}{2}}\left( \begin{array}{cc} -{\textsf{d}}u &  e^{u}\ell {\textsf{d}}x\\ e^{-u} \ell {\textsf{d}}x &  {\textsf{d}}u\end{array}\right) . \end{aligned}$$

Recall now the description of Fenchel–Nielsen flow, using pullback under (56). The corresponding $A_\tau$ is obtained by pullback:

$$\begin{aligned} A_\tau ={\frac{1}{2}}\left( \begin{array}{cc} -{\textsf{d}}u &  e^{u}(\ell {\textsf{d}}x+\tau f'(u)\ {\textsf{d}}u)\\ e^{-u}(\ell {\textsf{d}}x+\tau f'(u){\textsf{d}}u) &  {\textsf{d}}u\end{array}\right) . \end{aligned}$$

Note that $A_\tau$ agrees with *A* for $|u|\ge \epsilon$, hence it defines a new global connection $\theta _\tau$. Replacing $\tau$ with $\tau _t$, and taking a *t*-derivative, this gives the tangent vector

$$\begin{aligned} b={\frac{1}{2}}\left( \begin{array}{cc} 0&  e^{u}\\ e^{-u}&  0\end{array}\right) \dot{\tau }_0 f'(u)\,{\textsf{d}}u. \end{aligned}$$

The tangent vector corresponding to a change of the length parameter $\ell$ is obtained by replacing $\ell$ with $\ell _t$ and taking a *t*-derivative:

$$\begin{aligned} a={\frac{1}{2}}\left( \begin{array}{cc} 0&  e^{u}\\ e^{-u}&  0\end{array}\right) \dot{\ell }_0\ {\textsf{d}}x. \end{aligned}$$

We may arrange that *a* has this form on the collar neighborhood $|u|\le \epsilon$, but vanishes outside of a larger collar neighborhood (say, $|u|\le 2\epsilon$).

Hence, the Atiyah–Bott form on these tangent vectors evaluates to

$$\begin{aligned} \omega _{AB}(a,b)=\int _\Sigma {\text {tr}}(a b)=\frac{1}{2} \int _{|r|\le \epsilon } \dot{l}_0\dot{\tau }_0 {\textsf{d}}x\wedge f'(u) {\textsf{d}}u=\frac{1}{2} \dot{l}_0\dot{\tau }_0.\end{aligned}$$

This shows $\omega (\frac{\partial }{\partial \ell _i},\frac{\partial }{\partial \tau _i})=\frac{1}{2}$. On the other hand, if *a*, *b* are tangent vectors corresponding to twist or length deformations for *non-intersecting* geodesics, then $\omega _{AB}(a,b)=0$ since we may take the support of *a*, *b* to be disjoint. Thus, for example, $\omega (\frac{\partial }{\partial \ell _{i_1}},\frac{\partial }{\partial \ell _{i_2}})=0$ for $i_1\ne i_2$. Similarly, the pairing of the tangent vectors $\frac{\partial }{\partial \ell _{i}},\ \frac{\partial }{\partial \tau _{i}}$ with a tangent vector $\frac{\partial }{\partial \ell _j}$, corresponding to the change of length parameter for the *j*-th trumpet, is zero. It remains to check (62) on pairs of tangent vectors, one of which is a vector field $v\in {\text {Vect}}(\textsf{C}_j)$ on the ideal boundary of the *j*-th trumpet. For this, it suffices to observe that both sides satisfy the moment map condition

$\omega (v,\cdot )=\langle {\textsf{d}}L_j,v\rangle$ where $L_j$ is the Hill potential for the *j*th boundary. $\square$

### Darboux coordinates on the trumpet space

The expression (62) for the symplectic form on ${\text {Teich}}(\Sigma )$ involves the symplectic structure $\omega _N$ on the space $N={\text {Diff}}_\textsf{o}(S^1)\times \mathbb {R}_{>0}$ associated to the trumpet end. We may go one step further and introduce Darboux coordinates on the space *N*, and hence on ${\text {Teich}}(\Sigma )$.

Consider the symplectic structure on $\widetilde{N}=\mathbb {R}_{>0}\times \widetilde{{\text {Diff}}}_\textsf{o}(S^1)$, given by (60) with ${\textsf{F}}$ replaced by a lift $\widetilde{{\textsf{F}}}$ to the universal cover. We may regard $\widetilde{{\textsf{F}}}$ as $\mathbb {Z}$-equivariant function on $\mathbb {R}$, that is, $\widetilde{{\textsf{F}}}(x+1)=\widetilde{{\textsf{F}}}(x)+1$. The expression

63 $$\begin{aligned} u(x)= \log (\widetilde{{\textsf {F}}}'(x))+{\ell } (\widetilde{{\textsf {F}}}(x)-x) \end{aligned}$$

is a periodic function on $\mathbb {R}$. Taking into account the dependence on $\ell ,\widetilde{{\textsf{F}}}$, this is a function on $\widetilde{N}$ with values in $|\Omega |^0_{S^1}$. Thus ${\textsf{d}}u\wedge {\textsf{d}}u'$ is a 2-form on *N* with values in $|\Omega |^1_{S^1}$; integrating over $S^1$ it is a 2-form on $\widetilde{N}$.

#### Proposition 8.4

$$\begin{aligned} \omega _{\widetilde{N}}=-{\frac{1}{2}}{\textsf{d}}\ell \wedge {\textsf{d}}u_0+\frac{1}{4}\int _{S^1}\ {\textsf{d}}u\wedge {\textsf{d}}u' \end{aligned}$$

where $u_0=\int _{S^1} \ u$ (a scalar function of $(\ell ,\widetilde{\textsf{F}})\in \widetilde{N}$).

#### Proof

We work out the terms appearing in

$$\begin{aligned} \frac{1}{4}\int _{S^1}\ {\textsf{d}}u\wedge {\textsf{d}}u' =\frac{1}{4}\int _{S^1} {\textsf{d}}\big (\log (\widetilde{{\textsf{F}}}')+{\ell } (\widetilde{{\textsf{F}}}-{\text {Id}})\big )\wedge {\textsf{d}}\big ( \log (\widetilde{{\textsf{F}}}')+{\ell } (\widetilde{{\textsf{F}}}-{\text {Id}}) \big )' \end{aligned}$$

according to their homogeneity with respect to $\ell$, and compare to the corresponding terms in (61). The term of homogeneity 0 is $\frac{1}{4}(\widetilde{F'})^{-2}{\textsf{d}}\widetilde{F}'\wedge {\textsf{d}}\widetilde{F}''$, matching that in (61). The terms of homogeneity 1 are (using integration by parts to combine two terms)

$$\begin{aligned} {\frac{1}{2}}\int _{S^1} {\textsf {d}}\widetilde{F}'\wedge {\textsf {d}}\ell + {\frac{1}{2}}\int _{S^1} {\textsf {d}}\ell \wedge {\textsf {d}}\log (\widetilde{F}'). \end{aligned}$$

The first integral is zero, by the fundamental theorem of calculus, while the second integral gives one of the terms of ${\frac{1}{2}}{\textsf{d}}\ell \wedge {\textsf{d}}u_0$. The terms of homogeneity 2 are

$$\begin{aligned} =\frac{1}{4}\int _{S^1} \ell ^2 {\textsf{d}}\widetilde{{\textsf{F}}}\wedge {\textsf{d}}\widetilde{{\textsf{F}}}' +{\frac{1}{2}}\int _{S^1} (\widetilde{{\textsf{F}}}'-1){\textsf{d}}\widetilde{{\textsf{F}}}\wedge \ell {\textsf{d}}\ell \end{aligned}$$

(again we used an integration by parts to combine two terms). The integral

$$\begin{aligned} -\int _{S^1}{\textsf{d}}\widetilde{F}\wedge \ell {\textsf{d}}\ell ={\textsf{d}}\ell \wedge {\textsf{d}}\int _{S^1} \ell \widetilde{F} ={\textsf{d}}\ell \wedge {\textsf{d}}\int _{S^1} \ell (\widetilde{F}-{\text {Id}}) \end{aligned}$$

gives one of the terms in ${\textsf{d}}\ell \wedge {\textsf{d}}u_0$. The remaining terms match the corresponding terms in (61). $\square$

Having $\omega _{\widetilde{N}}$ in this form, it is straightforward to introduce Darboux coordinates, by its Fourier expansion:

$$\begin{aligned} u(x)=\sum _{n\in \mathbb {Z}} u_n e^{2\pi i nx}. \end{aligned}$$

We obtain:

#### Proposition 8.5

The symplectic form on $\widetilde{N}$ is given by

$$\begin{aligned} \omega _{\widetilde{N}}=-{\frac{1}{2}}{\textsf {d}}\ell \wedge {\textsf {d}}u_0+\pi i\sum _{m>0} m\ {\textsf {d}}u_{-m}\wedge {\textsf {d}}u_m. \end{aligned}$$

Rewriting the second sum in terms of real and imaginary parts of $u_m$, one obtains a Darboux normal form.

## Data Availability

No datasets were generated or analysed during the current study.

## Appendix A: Affine Moment Maps, Central Extensions

### A.1. Central extensions

Let *H* be a Lie group with Lie algebra $\mathfrak {h}$. Given a central extension

$$\begin{aligned} 0\rightarrow \mathbb {R}\rightarrow \widehat{\mathfrak {h}}\rightarrow \mathfrak {h}\rightarrow 0, \end{aligned}$$

let $\mathcal {E}\subseteq \widehat{\mathfrak {h}}^*$ be the affine space of linear functionals taking $1\in \mathbb {R}$ to 1. The adjoint action of *H* on $\widehat{\mathfrak {h}}$ defines an affine *H*-action on this space, with linear part the coadjoint action on $\mathfrak {h}^*$. Conversely, suppose $\mathcal {E}$ is an affine space with an affine *H*-action, with underlying linear *H*-space the coadjoint representation. For $\mu \in \mathcal {E}$, the map $\mathfrak {h}\rightarrow \mathfrak {h}^*,\ \xi \mapsto \xi . \mu$, defined by the infinitesimal action, is a Lie algebra cocycle. Suppose this cocycle is skew-symmetric:

64 $$\begin{aligned} \langle \xi . \mu ,\ \eta \rangle =-\langle \eta . \mu ,\ \xi \rangle \end{aligned}$$

for all $\xi ,\eta$. (This condition does not depend on the choice of $\mu$.) Then one obtains a central extension: Take $\widehat{\mathfrak {h}}$ to be the vector space of affine-linear functions on $\mathcal {E}$, with bracket

$$\begin{aligned} [\widehat{\xi },\widehat{\eta }](\mu )=\langle \xi . \mu ,\ \eta \rangle . \end{aligned}$$

Here $\xi ,\eta$ are the linear functionals underlying $\widehat{\xi },\widehat{\eta }\in \widehat{\mathfrak {h}}$. The affine space $\mathcal {E}$ is a Poisson submanifold of $\widehat{\mathfrak {h}}^*$, with symplectic leaves the coadjoint orbits. The action groupoid $H\times \mathcal {E}\rightrightarrows \mathcal {E}$ has a canonical symplectic structure making it into a symplectic groupoid. See [3, Example A.12] for further discussion.

### A.2. Affine moment maps

Let $\mathcal {E}$ be an affine *H*-space as above, with underlying linear *H*-space the coadjoint representation. Given an *H*-manifold *M* with a closed invariant 2-form $\omega$, we may consider $\mathcal {E}$-valued affine moment maps

$$\begin{aligned} \Phi :M\rightarrow \mathcal {E}; \end{aligned}$$

that is, $\Phi$ is *H*-equivariant and satisfies the moment map condition^4^

$$\begin{aligned} \omega (\xi _M,\cdot )=-\langle {\textsf{d}}\Phi ,\xi \rangle . \end{aligned}$$

Note that the differential ${\textsf{d}}\Phi$ is a 1-form with values in the linear space $\mathfrak {h}^*$, hence its pairing with $\xi$ is defined. Examples of Hamiltonian *H*-spaces with $\mathcal {E}$-valued moment maps are the coadjoint orbits in $\mathcal {E}$, with the KKS symplectic structure.

A necessary condition for the existence of an affine moment map (for some $\mathcal {E}$) is that the 1-forms $\alpha \in \Omega ^1(M,\mathfrak {h}^*)$, given as $\langle \alpha ,\xi \rangle =-\omega (\xi _M,\cdot )$, are exact. In fact, this condition is sufficient as well:

#### Proposition A.1

Let *M* be a connected *H*-manifold, with an invariant closed 2-form $\omega \in \Omega ^2(M)$. Suppose that the 1-form

$$\begin{aligned} \alpha \in \Omega ^1(M,\mathfrak {h}^*)\end{aligned}$$

given as $\langle \alpha ,\xi \rangle =\omega (\xi _M,\cdot )$ is exact. Let $\mathcal {E}$ be the affine space of all primitives of $\alpha$, and let

$$\begin{aligned} \Phi :M\rightarrow \mathcal {E}\end{aligned}$$

be the map taking any point of *M* to the unique primitive vanishing at that point. Then $\mathcal {E}$ is an affine *H*-space, with underlying linear action the coadjoint action, satisfying the skew-symmetry (64). Furthermore, $\Phi$ is an affine moment map.

#### Proof

Since *M* is connected, a primitive of $\alpha$ is unique up to a constant function with values in $\mathfrak {h}^*$. This shows that $\mathcal {E}$ is an affine space over $\mathfrak {h}^*$. The group *H* acts on $\mathcal {E}\subseteq C^\infty (M,\mathfrak {h}^*)$ by $(h. f)(m)= {\text {Ad}} _h \big (f(h^{-1}. m)\big )$; hence the difference of two elements transforms under the coadjoint action. For $f\in \mathcal {E}$ we have

$$\begin{aligned} \langle \xi .f,\eta \rangle =\langle \mathcal {L}_{\xi _M} f+{\text {ad}}_\xi f,\eta \rangle =-\omega (\xi _M,\eta _M) -\langle f,[\xi ,\eta ]\rangle , \end{aligned}$$

which is skew-symmetric in $\xi ,\eta$. To verify that $\Phi$ (as in the proposition) is a moment map, fix $m_0\in M$. Then $\Phi (m_0)(m)=\int _{m_0}^m \alpha =-\Phi (m)(m_0)$. Hence, the map $m\mapsto \Phi (m)|_{m_0}$ is a primitive for $-\alpha$. This shows $\langle {\textsf{d}}\Phi ,\xi \rangle =-\alpha (\xi )=-\omega (\xi _M,\cdot )$. $\square$

Note that $\mathcal {E}$, and hence the central extension of $\mathfrak {h}$, is determined by the pullback of $\omega$ to any *G*-invariant submanifold of *M*. For example, if the action has a fixed point, or more generally if it admits an invariant isotropic submanifold, then the central extension is trivial, and the action admits an ordinary $\mathfrak {h}^*$-valued moment map.

The constructions above apply to infinite-dimensional settings, provided that one has a reasonable notion of smooth dual. In particular, the affine action of ${\text {Diff}}_+(\textsf{C})$ on the space ${\text {Hill}}(\textsf{C})$ of Hill operators (Sect. 7.1) defines a central extension of ${\text {Vect}}(\textsf{C})$, the Virasoro algebra.

## Appendix B: Gauge Theory Constructions

In this “Appendix”, we review the Atiyah–Bott construction for principal *G*-bundles $P\rightarrow \Sigma$ over oriented surfaces with boundary. Here *G* is any Lie group with an invariant inner product on its Lie algebra (we are mainly interested in the case $G={\text {PSL}}(2,\mathbb {R})$).

### B.1. Atiyah algebroid, connections

Let $P\rightarrow M$ be a principal *G*-bundle. The groups of gauge transformations and automorphisms are denoted ${\text {Gau}} (P)\subseteq {\text {Aut}} (P)$. The *Atiyah algebroid* ${\text {At}}(P)=TP/G\rightarrow M$ is the Lie algebroid whose sections are the *G*-invariant vector fields on *P*, that is, infinitesimal automorphisms. It fits into the exact sequence of Lie algebroids

65 $$\begin{aligned} 0\longrightarrow \mathfrak {g}(P)\longrightarrow {\text {At}} (P) {\mathop {\longrightarrow }\limits ^{\textsf{a}}} TM\longrightarrow 0, \end{aligned}$$

with the adjoint bundle $\mathfrak {g}(P)=P\times _G \mathfrak {g}$. On the level of sections, this is the exact sequence $0\rightarrow \mathfrak {gau} (P)\rightarrow \mathfrak {aut} (P)\rightarrow {\text {Vect}}(M)\rightarrow 0$. A principal connection $\theta \in \Omega ^1(P,\mathfrak {g})^G$ is equivalent to a vector bundle splitting of the Atiyah sequence. This may be described by either of the bundle maps

$$\begin{aligned} s^\theta : {\text {At}} (P)\rightarrow \mathfrak {g}(P),\ \ \ j^\theta :TM\rightarrow {\text {At}} (P) \end{aligned}$$

called *vertical projection* and *horizontal lift*; thus $s^\theta |_{\mathfrak {g}(P)}={\text {id}}_{\mathfrak {g}(P)}$, $\textsf{a}\circ j^\theta ={\text {id}}_{TM},\ s^\theta \circ j^\theta =0$. The section $s^\theta (v)$ corresponds to $\iota _v\theta$ under the identification $\mathfrak {g}(Q)\cong \Omega ^0(Q,\mathfrak {g})^G$. We denote by

$$\begin{aligned} {\textsf{d}}^\theta :\Omega ^p(M,\mathfrak {g}(P))\rightarrow \Omega ^{p+1}(M,\mathfrak {g}(P)) \end{aligned}$$

the covariant derivative; in terms of the identification of forms $\beta \in \Omega ^\bullet (M,\mathfrak {g}(P))$ with *G*-basic forms $\widetilde{\beta }\in \Omega ^\bullet (P,\mathfrak {g})$ we have $\widetilde{{\textsf{d}}^\theta \beta }={\textsf{d}}\widetilde{\beta }+[\theta ,\widetilde{\beta }]$. The curvature $F^\theta \in \Omega ^2(M,\mathfrak {g}(P))$ corresponds to the basic form $\widetilde{F}^\theta ={\textsf{d}}\theta +{\frac{1}{2}}[\theta ,\theta ]$.

The set $\mathcal {A}(P)$ of principal connections is an affine space over $\Omega ^1(M,\mathfrak {g}(P))$: given a smooth family of connections $\theta _t$ with $\theta _0=\theta$, the corresponding tangent vector $\beta \in \Omega ^1(M,\mathfrak {g}(P))$ is determined by either one of the equations

$$\begin{aligned} \frac{d}{d t}|_{t=0}\theta _t=\widetilde{\beta },\ \ \ \frac{d}{d t}|_{t=0}s^{\theta _t}=\beta \circ \textsf{a},\ \ \ \frac{d}{d t}|_{t=0}j^{\theta _t}=-\beta . \end{aligned}$$

#### Lemma B.1

The generating vector fields for the ${\text {Aut}} (P)$-action on $\mathcal {A}(P)$ are given by

66 $$\begin{aligned} v_{\mathcal {A}(P)}|_\theta =-{\textsf{d}}^\theta (s^\theta (v))-\iota _{\textsf{a}(v)}F^\theta ,\ \ v\in \mathfrak {aut}(P)=\Gamma ( {\text {At}} (P)). \end{aligned}$$

In particular, for $v\in \Gamma (\mathfrak {g}(P))=\Omega ^0(M,\mathfrak {g}(P))$, the generating vector field is $-{\textsf{d}}^\theta v$.

#### Proof

By our sign convention for generating vector fields, $v_{\mathcal {A}(P)}|_\theta =-v.\theta$. Regarding *v* as a *G*-invariant vector field $\widetilde{v}$ on *P*, we have

$$\begin{aligned} \widetilde{v.\theta }=\mathcal {L}_{\widetilde{v}}\theta ={\textsf{d}}\iota _{\widetilde{v}}\theta +\iota _{\widetilde{v}}{\textsf{d}}\theta = ({\textsf{d}}+[\theta ,\cdot ]) \iota _{\widetilde{v}}\theta +\iota _{\widetilde{v}} \widetilde{F}^\theta =\widetilde{{\textsf{d}}^\theta (s^\theta (v))}+\widetilde{\iota _{\textsf{a}(v)}F^\theta }. \end{aligned}$$

$$\square $$

### B.2. Trivializations

Suppose $P\rightarrow M$ admits a section $\iota :M\rightarrow P$, defining a trivialization $P\cong M\times G$ such that $\iota (m)=(m,e)$ with the principal action $a. (m,g)=(m,ga^{-1})$. Connections $\theta \in \mathcal {A}(P)$ are described in terms of their connection 1-forms $A=\iota ^*\theta$ by

$$\begin{aligned} \theta = {\text {Ad}} _{g^{-1}}(A)+{\text {pr}}_2^*\theta ^L\end{aligned}$$

The trivial connection (given by $\theta ={\text {pr}}_2^*\theta ^L$) gives a splitting of the Atiyah algebroid ${\text {At}} (P)\cong TM\oplus \mathfrak {g}(P)$. The trivialization of *P* determines a trivialization of all its associated bundles, and in particular gives an isomorphism

67 $$\begin{aligned} \mathfrak {g}(P)\cong M\times \mathfrak {g}. \end{aligned}$$

We have

$$\begin{aligned} s^\theta (X,\xi )=\xi +\iota _X A,\ \ j^\theta (X)=(X,-\iota _X A). \end{aligned}$$

The covariant derivative is the operator on $\Omega ^\bullet (M,\mathfrak {g}(P))\cong \Omega ^\bullet (M,\mathfrak {g})$ given by ${\textsf{d}}_A={\textsf{d}}+[A,\cdot ]$; the curvature is $F_A=\iota ^* \widetilde{F}^\theta ={\textsf{d}}A+{\frac{1}{2}}[A,A]$.

#### Remark B.2

(Signs, I) We stress that the isomorphism

68 $$\begin{aligned} \mathfrak {gau} (P)=C^\infty (M,\mathfrak {g}) \end{aligned}$$

given by (67) differs by sign from the ‘standard’ identification as the Lie algebra of ${\text {Gau}} (P)=C^\infty (M,G)$ (with pointwise multiplication). In fact, (68) takes a function $\xi \in C^\infty (M,\mathfrak {g})$ to the vertical vector field whose restriction to $\{m\}\times G$ is $\xi (m)^R$. In particular, (68) induces *minus* the pointwise Lie bracket on $C^\infty (M,\mathfrak {g})$. More generally, $\mathfrak {aut}(P)=C^\infty (M,\mathfrak {g})\rtimes {\text {Vect}}(M)$ with the bracket

$$\begin{aligned} [(\xi ,X),(\eta ,Y)]=(-[\xi ,\eta ],\mathcal {L}_X\eta -\mathcal {L}_Y\xi ). \end{aligned}$$

### B.3. Central extensions

Suppose $Q\rightarrow \textsf{C}$ is a principal *G*-bundle over a compact oriented 1-manifold, and that the Lie algebra $\mathfrak {g}$ carries an invariant metric (denoted by a dot). Then $\mathfrak {g}(Q)$ inherits a bundle metric. The affine space $\mathcal {A}(Q)$ of connections has $\mathfrak {gau} (Q)^*=\Omega ^1(\textsf{C},\mathfrak {g}(Q))$ as its underlying linear space, where the pairing with $\mathfrak {gau} (Q)=\Omega ^0(\textsf{C},\mathfrak {g}(Q))$ is given by the metric followed by integration. This identification takes the linear part of the gauge action to the coadjoint action. Furthermore, $\langle \xi .\theta ,\eta \rangle =-\langle {\textsf{d}}^\theta \xi ,\eta \rangle$ satisfies the skew-symmetry condition (64). By the discussion of “Appendix A.1”, this defines a central extension of $\mathfrak {gau} (Q)$.

#### Remark B.3

(Signs, II) For trivial bundles $Q=\textsf{C}\times G$, one often uses the ‘standard’ identification $\mathfrak {gau} (Q)\cong C^\infty (\textsf{C},\mathfrak {g})$ to define the pairing. As explained in Remark B.2, this is opposite to the identification coming from $\mathfrak {g}(Q)\cong \textsf{C}\times \mathfrak {g}$, and hence results in the opposite pairing.

We may also consider the larger group ${\text {Aut}} (Q)$ of all principal bundle automorphisms, with Lie algebra $\mathfrak {aut} (Q)=\Gamma ( {\text {At}} (Q))$; its smooth dual is $\mathfrak {aut} (Q)^*=\Omega ^1(\textsf{C}, {\text {At}} (Q)^*)$. Let

69 $$\begin{aligned} \mathcal {E}(Q)\subseteq \Gamma ({\text {Sym}}^2 {\text {At}} (Q)^*) \end{aligned}$$

be the affine space of all fiberwise quadratic forms on ${\text {At}} (Q)$ whose restriction to $\mathfrak {g}(Q)$ is given by $\xi \mapsto {\frac{1}{2}}\xi \cdot \xi$. The underlying linear space consists of quadratic forms on ${\text {At}} (Q)$ whose restriction to $\mathfrak {g}(Q)$ is zero; it is identified with the space $\Omega ^1(\textsf{C}, {\text {At}} (Q)^*)$, where an element $\gamma$ of this space defines the quadratic form $w\mapsto \langle \gamma (\textsf{a}(w)),w\rangle$. The group ${\text {Aut}} (Q)$ acts on $\mathcal {E}(Q)$, and the underlying linear action is coadjoint action. One may also check that it satisfies the skew-symmetry property (64); hence one obtains a central extension of $\mathfrak {aut} (Q)$.

#### Remark B.4

There is a natural map

$$\begin{aligned} \mathcal {E}(Q)\rightarrow \mathcal {A}(Q),\ \ \phi \mapsto \theta \end{aligned}$$

given by $s^\theta (v)\cdot \xi =-2\phi (v,\xi )$ for $v\in \Gamma ( {\text {At}} (Q),\ \xi \in \mathfrak {g}(Q)$ (where we think of $\phi$ as a symmetric bilinear form). This map is affine with respect to the quotient map $\Omega ^1(\textsf{C}, {\text {At}} (Q)^*)\rightarrow \Omega ^1(\textsf{C},\mathfrak {g}(Q)^*)$, and determines a Lie algebra morphism $\widehat{ \mathfrak {gau} }(Q)\rightarrow \widehat{ \mathfrak {aut} }(Q)$ lifting the natural inclusion.

#### Remark B.5

There is a natural ${\text {Aut}} (Q)$-equivariant section section

70 $$\begin{aligned} \mathcal {A}(Q)\rightarrow \mathcal {E}(Q),\ \ \theta \mapsto {\frac{1}{2}}s^\theta {\mathop {\vee }\limits ^{.}} s^\theta . \end{aligned}$$

Here $\vee$ denotes the product in the symmetric algebra. The map (70) is the moment map for the ${\text {Aut}} (Q)$-action on $\mathcal {A}(Q)$, in the sense that it restricts to moment maps on the coadjoint orbits $\mathcal {O}\subseteq \mathcal {A}(Q)$.

For a trivial bundle $Q=\textsf{C}\times G$, we have $\mathcal {A}(Q)=\Omega ^1(\textsf{C},\mathfrak {g})$ and $\mathcal {E}(Q)=\Omega ^1(\textsf{C},\mathfrak {g})\times |\Omega |^2_\textsf{C}$; in terms of these identifications the map is

$$\begin{aligned} A\mapsto (A,{\frac{1}{2}}A\cdot A). \end{aligned}$$

It is gauge equivariant for the action $h.(A,q)=(h\bullet A,\,q+A\cdot h^*\theta ^L-{\frac{1}{2}}h^*\theta ^L\cdot h^*\theta ^L$).

### B.4. Atiyah–Bott

We now assume that $\Sigma$ is a compact oriented surface, possibly with boundary, and that the Lie algebra $\mathfrak {g}={\text {Lie}}(G)$ comes with an invariant metric. Let $P\rightarrow \Sigma$ be a principal *G*-bundle.

#### Definition B.6

The *Atiyah–Bott form* on $\mathcal {A}(P)$ is given by

$$\begin{aligned} \omega _{AB}(a,b)=\int _{\Sigma } a\cdot b,\ \ \ \ a,b\in \Omega ^1(\Sigma ,\mathfrak {g}(P)).\end{aligned}$$

The Atiyah–Bott form $\omega _{AB}$ is closed, by translation invariance, and nondegenerate in the weak sense that $\omega _{AB}(a,\cdot )=0 \Leftrightarrow a=0$. The 2-form is invariant under the action of the group $^b \!\! {\text {Aut}} _+(P)$ of principal bundle automorphisms whose base map lies in $^b {\text {Diff}}_+(\Sigma )$. We are interested in a moment map for this action. The Lie algebra $^b \!\!\mathfrak {aut}(P)$ consists of *G*-invariant vector fields on *P* that are tangent to $\partial P$; these are the sections of a Lie algebroid $^b \!\! {\text {At}} (P)$. Let $\partial P=P|_{\partial \Sigma }$ the boundary restriction of *P*. For a connection $\theta$, denote by $\partial \theta$ its pullback to a connection on *P*.

#### Proposition B.7

The action of $^b \!\! {\text {Aut}} _+(P)$ on $\mathcal {A}(P)$ has moment map

$$\begin{aligned} \mathcal {A}(P)\rightarrow \Omega ^2(\Sigma , {\text {At}} (P)^*)\times \mathcal {E}(\partial P),\ \theta \mapsto (-F^\theta \cdot s^\theta ,{\frac{1}{2}}s^{\partial \theta }{\mathop {\vee }\limits ^{.}} s^{\partial \theta }). \end{aligned}$$

For the action of ${\text {Gau}} (P)$, the moment map takes values in $\Omega ^2(\Sigma ,\mathfrak {g}(P))\times \mathcal {A}(\partial P)$, and is given by $(-F^\theta ,\partial \theta )$.

#### Proof

Let $v\in  ^b \mathfrak {aut} (P)=\Gamma ( ^b \!\! {\text {At}} (P))$, with boundary restriction $\partial v$. Given $b\in T_\theta \mathcal {A}(P)=\Omega ^1(\Sigma ,\mathfrak {g}(P))$, we have, using (66),

$$\begin{aligned} \omega _{AB}(v^\sharp |_\theta ,\, b)&= \int _\Sigma v^\sharp |_\theta \cdot b\\  &=-\int _\Sigma \iota _{\textsf {a}(v)}F^\theta \cdot b-\int _\Sigma {\textsf {d}}^\theta (s^\theta (v))\cdot b\\  &=\int _\Sigma \iota _{\textsf {a}(v)} b\cdot F^\theta + \int _\Sigma s^\theta (v)\cdot {\textsf {d}}^\theta b-\int _{\partial {\Sigma }} s^{\partial \theta }(\partial v)\cdot \partial b, \end{aligned}$$

where $\partial b\in T_{\partial \theta }\mathcal {A}(\partial P)$ is the pullback of *b* to the boundary. Suppose $b\in T_\theta \mathcal {A}(P)$ is realized as the velocity vector for a family of connections $\theta _t$ with

$$\begin{aligned} \theta _0=\theta ,\ \ \ \frac{d}{d t}\Big |_{t=0}\theta _t=\widetilde{b}. \end{aligned}$$

Then $\frac{d}{d t}\big |_{t=0} s^{\theta _t}=b\circ \textsf{a},\ \frac{d}{d t}\big |_{t=0} F^{\theta _t}={\textsf{d}}^{\theta _t}b$, and therefore

$$\begin{aligned} \frac{d}{d t}\Big |_{t=0} F^{\theta _t}\cdot s^{\theta _t} = (b\circ \textsf{a})\cdot F^\theta +s^\theta \cdot {\textsf{d}}^\theta b.\end{aligned}$$

Here $b\circ \textsf{a},\ s^\theta$ are regarded as sections of ${\text {At}} (P)^*\otimes \mathfrak {g}(P)$, while $F^\theta ,\ {\textsf{d}}^\theta b$ are elements of $\Omega ^2(\Sigma ,\mathfrak {g}(P))$. For the boundary term, we note

$$\begin{aligned} \frac{d}{d t}\Big |_{t=0} \left( {\frac{1}{2}}s^{\partial \theta _t}{\mathop {\vee }\limits ^{.}} s^{\partial \theta _t}\right) =s^{\partial \theta }{\mathop {\vee }\limits ^{.}}(\partial b\circ \textsf{a}) =s^{\partial \theta }\cdot \partial b \end{aligned}$$

where the last equality comes from the inclusion $\Omega ^1(\partial {\Sigma }, {\text {At}} (\partial P)^*)\hookrightarrow \Gamma ({\text {Sym}}^2( {\text {At}} (\partial P)^*))$. This gives

$$\begin{aligned} \omega _{AB}(v^\sharp |_\theta ,\, b)= \frac{d}{d t}\Big |_{t=0} \int _\Sigma F^{\theta _t}\cdot s^{\theta _t}(v)-\int _{\partial {\Sigma }} \Big ( \frac{d}{d t}\Big |_{t=0} {\frac{1}{2}}s^{\partial \theta _t}\vee s^{\partial \theta _t}\Big ) (\partial v,\cdot ) \Big ). \end{aligned}$$

$$\square $$

#### Remark B.8

(Signs, III) The sign in the moment map for ${\text {Gau}} (P)$ depends on the identifications $\mathfrak {gau} (P)\cong \Omega ^2(P,\mathfrak {g}(P))$ and $\mathfrak {gau} (\partial P)\cong \Omega ^1(\partial P,\mathfrak {g}(\partial P))$. For trivial bundles, one often uses the opposite pairing, resulting in a sign change of the moment map. See Remarks B.2, B.3.

## Appendix C: The Trumpet Moduli Space as a Symplectic Cross-Section

Our starting point will be the interpretation of *N* as a symplectic slice in a symplectic groupoid $\mathcal {G}\rightrightarrows {\text {Hill}}(S^1)$, see [3]. As a groupoid, $\mathcal {G}\cong {\text {Hill}}(S^1)\times {\text {Diff}}_\textsf{o}(S^1)$ (an action groupoid), with source and target maps $\textsf{s}(T,{\textsf{F}})=T,\ \textsf{t}(T,{\textsf{F}})={\textsf{F}}. T$ and groupoid multiplication $(T_1,{\textsf{F}}_1)\circ (T_2,{\textsf{F}}_2)=(T_2,{\textsf{F}}_1\circ {\textsf{F}}_2)$. In terms of this ‘left trivialization’, the symplectic structure $\omega _\mathcal {G}$ is given by [3, Equation 47]

71 $$\begin{aligned} \omega _\mathcal {G}=\int _{S^1} \Big ( {\textsf{d}}T\wedge \frac{{\textsf{d}}\textsf{F}}{\textsf{F}'}+T\, \frac{{\textsf{d}}\textsf{F}}{\textsf{F}'}\wedge \big (\frac{{\textsf{d}}\textsf{F}}{\textsf{F}'}\big )'-\frac{1}{4} \big (\frac{{\textsf{d}}\textsf{F}}{\textsf{F}'}\big )'''\wedge \big (\frac{{\textsf{d}}\textsf{F}}{\textsf{F}'}\big )\Big ).\ \end{aligned}$$

For our description of the 2-form for the trumpet, it will be more convenient to work with ‘right trivialization’, expressing the 2-form in terms of $(T_0,{\textsf{F}})$ where

72 $$\begin{aligned} T={\textsf{F}}^{-1}. T_0=({\textsf{F}}')^2 T_0+{\frac{1}{2}}\mathcal {S}({\textsf{F}}). \end{aligned}$$

### Proposition C.1

In right trivialization,

$$\begin{aligned} \omega _\mathcal {G}={\textsf {d}}\int _{S^1}\ \ \Big ( T_0 {\textsf {F}}' -\frac{1}{4}({\textsf {F}}')^{-1}{\textsf {d}}{\textsf {F}}''\Big ). \end{aligned}$$

### Proof

This is based on a straightforward but lengthy calculation, substituting (72) and simplifying. Here are some relevant steps. Using the formula for the exterior differential of the Schwarzian derivative ([1, Lemma A.2]) one shows

$$\begin{aligned} \int _{S^1}\ {\textsf {d}}\mathcal {S}({\textsf {F}})\wedge \big (\frac{{\textsf {d}}\textsf {F}}{\textsf {F}'}\big )= \int _{S^1}\ \frac{{\textsf {d}}{\textsf {F}}'\wedge {\textsf {d}}{\textsf {F}}''}{(\textsf {F}')^2}. \end{aligned}$$

This then implies

$$\begin{aligned} \int _{S^1}\ {\textsf {d}}T\wedge \frac{{\textsf {d}}\textsf {F}}{\textsf {F}'} =\int _{S^1}\ \Big ({\textsf {F}}' {\textsf {d}}T_0\wedge {\textsf {d}}\textsf {F}+2T_0\,{\textsf {d}}{\textsf {F}}'\wedge {\textsf {d}}{\textsf {F}}+\frac{1}{2} \frac{{\textsf {d}}{\textsf {F}}'\wedge {\textsf {d}}{\textsf {F}}''}{(\textsf {F}')^2}\Big ). \end{aligned}$$

Furthermore,

$$\begin{aligned} \int _{S^1}\ T\, \frac{{\textsf {d}}\textsf {F}}{\textsf {F}'}\wedge \big (\frac{{\textsf {d}}\textsf {F}}{\textsf {F}'}\big )'=\int _{S^1}\ \Big (T_0\,{\textsf {d}}{\textsf {F}}'\wedge {\textsf {d}}{\textsf {F}}+\frac{1}{2} \mathcal {S}({\textsf {F}})\frac{{\textsf {d}}{\textsf {F}}\wedge {\textsf {d}}{\textsf {F}}'}{(\textsf {F}')^2}\Big ), \end{aligned}$$

and

$$\begin{aligned} -\frac{1}{4} \int _{S^1}\ \big (\frac{{\textsf{d}}\textsf{F}}{\textsf{F}'}\big )'''\wedge \big (\frac{{\textsf{d}}\textsf{F}}{\textsf{F}'}\big ) = \int _{S^1}\ \Big (-\frac{1}{4} \frac{{\textsf{d}}{\textsf{F}}'\wedge {\textsf{d}}{\textsf{F}}''}{(\textsf{F}')^2} -\frac{1}{2} \mathcal {S}({\textsf{F}})\frac{{\textsf{d}}{\textsf{F}}\wedge {\textsf{d}}{\textsf{F}}'}{(\textsf{F}')^2} \Big ). \end{aligned}$$

Adding these three contributions, the formula for $\omega _\mathcal {G}$ follows.$\square$

Passage to the slice $N=\mathbb {R}_{>0}\times {\text {Diff}}_\textsf{o}(S^1)$ amounts to putting $T_0(x)=-\frac{\ell ^2}{4}$, resulting in the formula (60).

## Footnotes

1 A *morphism of manifolds with boundary* $F:M_1\rightarrow M_2$ is a smooth map with the property that the pullback of a boundary defining function on $M_2$ is a boundary defining function on $M_1$. Note that such a map determines a morphism of the 0-tangent bundles.

2 In [3], we worked with the bundle *E* instead of $E^*$. The expression in (49) is therefore minus the transpose of that used in [3].

3 With our sign conventions, the flow of the vector field $f\,\frac{\partial }{\partial x}$ is of the form $F_t(x)=x-t f(x)+O(t^2)$. An additional sign arises comes from the choice of identification $\mathfrak {gau} (Q)\cong C^\infty (\textsf{C},\mathfrak {g})$ used in “Appendix B”.
